# A nested high-resolution unstructured grid 3-D ocean-sea ice-ice shelf setup for numerical investigations of the Petermann ice shelf and fjord

**DOI:** 10.1016/j.mex.2022.101668

**Published:** 2022-03-18

**Authors:** Abhay Prakash, Qin Zhou, Tore Hattermann, Weiyang Bao, Rune Graversen, Nina Kirchner

**Affiliations:** a Department of Physical Geography, Stockholm University, Stockholm 10691, Sweden; b Bolin Centre for Climate Research, Stockholm University, Stockholm 10691, Sweden; c Akvaplan-niva, Tromsø 9296, Norway; d Norwegian Polar Institute, Tromsø 9296, Norway; e Department of Physics and Technology, University of Tromsø, Tromsø 9019, Norway; f School of Marine Sciences and Policy, University of Delaware, Newark, DE 19716, USA; g Norwegian Meteorological Institute, Tromsø 9293, Norway

**Keywords:** FVCOM, Numerical modeling, Ice shelf-ocean interactions, Greenland, Outlet glacier

## Abstract

Three-dimensional numerical simulation of circulation in fjords hosting marine-terminating ice shelves is challenging because of the complexity of processes involved in such environments. This often requires a comprehensive model setup. The following elements are needed: bathymetry (usually unknown beneath the glacier tongue), ice shelf draft (impacting water column thickness), oceanographic state (including tidal elevation, salinity, temperature and velocity of the water masses), sea ice and atmospheric forcing. Moreover, a high spatial resolution is needed, at least locally, which may be augmented with a coarser and computationally cheaper (nested) model that provides sufficiently realistic conditions at the boundaries. Here, we describe procedures to systematically create such a setup that uses the Finite Volume Community Ocean Model (FVCOM) for the Petermann Fjord, Northwest Greenland. The first simulations are validated against temperature and salinity observations from the Petermann Fjord in September 2019. We provide

• Complete bathymetry, ice-draft and water column thickness datasets of the Petermann Fjord, with an improved representation of the topography underneath the glacier tongue.
• Boundary conditions for ocean, atmosphere and sea ice derived from a suite of high-resolution regional models that can be used to initialize and run the regional ocean model with realistic geophysical settings.

## Graphical abstract

**Figure:**
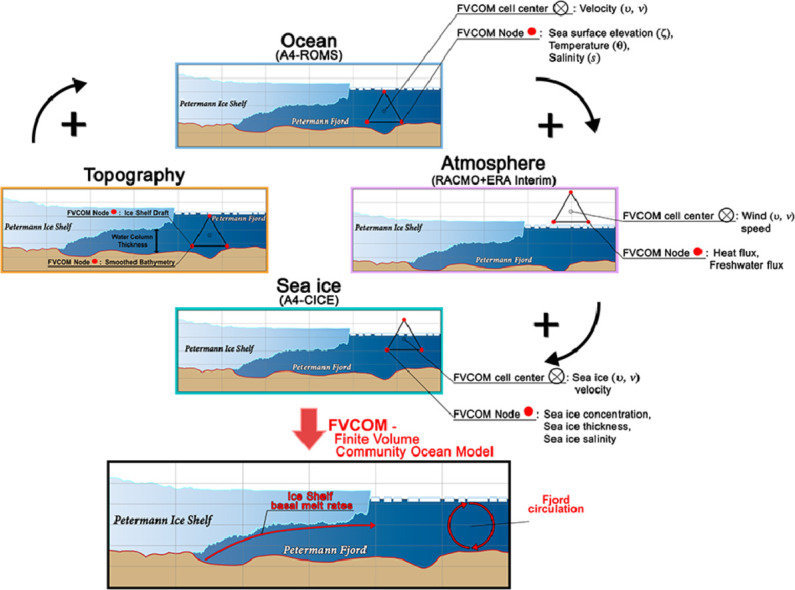

Specifications table

**Table:** 

Subject Area;	Earth and Planetary Sciences
More specific subject area;	Glaciology, Physical Oceanography, Numerical Modelling
Method name;	Setup of an FVCOM simulation centered on the Petermann Fjord, Northwest Greenland
Name and reference of original method;	**FVCOM:**C. Chen, H. Liu, R.C. Beardsley, An unstructured grid, finite-volume, three-dimensional, primitive equations ocean model: application to coastal ocean and estuaries, J. Atmos. Ocean. Technol. 20 (1) (2003) 159–186. C. Chen, R.C. Beardsley, G. Cowles, J. Qi, Z. Lai, G. Gao, D. Stuebe, Q. Xu, P. Xue, J. Ge, R. Ji, S. Hu, R. Tian, H. Huang, L. Wu, H. Lin, Y. Sun, L. Zhao. An Unstructured Grid, Finite-Volume Community Ocean Model FVCOM User Manual, SMAST/UMASSD Technical Report 13-0701, School of Marine Science and Technology. University of Massachusetts-Dartmouth, New Bedford, MA, USA (2013). **Ice shelf module:**Q. Zhou, T. Hattermann, Modeling ice shelf cavities in the unstructured-grid, finite volume community ocean model: implementation and effects of resolving small-scale topography, Ocean Modell. 146 (2020) 101536, doi:10.1016/j.ocemod.2019.101536. **4-km pan-Arctic (A4) ROMS model:**T. Hattermann, P.E. Isachsen, W.J. von Appen, J. Albretsen, A. Sundfjord, Eddy driven recirculation of Atlantic water in Fram Strait, Geophys. Res. Lett. 43 (2016) 3406–3414, doi:10.1002/2016GL068323. **AOTIM:**L. Padman, S. Erofeeva, A barotropic inverse tidal model for the Arctic Ocean, Geophys. Res. Lett. 31 (2004) L02303, doi:10.1029/2003GL019003. **RACMO 2.3p2:**B. Noël, W. J. van de Berg, J. M. van Wessem, E. van Meijgaard, D. van As, J. T. M. Lenaerts, S. Lhermitte, P. Kuipers Munneke, C. J. P. P. Smeets, L. H. van Ulft, R. S. W. van de Wal, M. R. van den Broeke, Modelling the climate and surface mass balance of polar ice sheets using RACMO2, Part 1: Greenland (1958-2016). Cryosphere 12, 811-831 (2018). **ERA-Interim (1979-2017):** D. P. Dee, S. M. Uppala, A. J. Simmons, P. Berrisford, P. Poli, S. Kobayashi, U. Andrae, M. A. Balmaseda, G. Balsamo, P. Bauer, P. Bechtold, A. C. M. Beljaars, L. van de Berg, J. Bidlot, N. Bormann, C. Delsol, R. Dragani, M. Fuentes, A. J. Geer, L. Haimberger, S. B. Healy, H. Hersbach, E. V. Hólm, L. Isaksen, P. Kållberg, M. Köhler, M. Matricardi, A. P. McNally, B. M. Monge- Sanz, J.-J. Morcrette, B.-K. Park, C. Peubey, P. de Rosnay, C. Tavolato, J.-N. Thépaut, F. Vitart, The ERA-Interim reanalysis: Configuration and performance of the data assimilation system. Q. J. R. Meteorol. Soc. 137, 553-597 (2011).
Resource availability;	The open source code Finite Volume Community Ocean Model (FVCOM) version 4.0 [Chen et al., 2003; Chen et al., 2013] is used in this study. The implementation of ice shelf cavities into FVCOM version 4.0 follows from Zhou and Hattermann, 2020. All model input (mesh, topography etc.), initial condition, nesting and forcing files that are required to initialize and run the numerical simulation will be made available upon request in appropriate format (netcdf or dat).

## Introduction

**Fig. 1:**
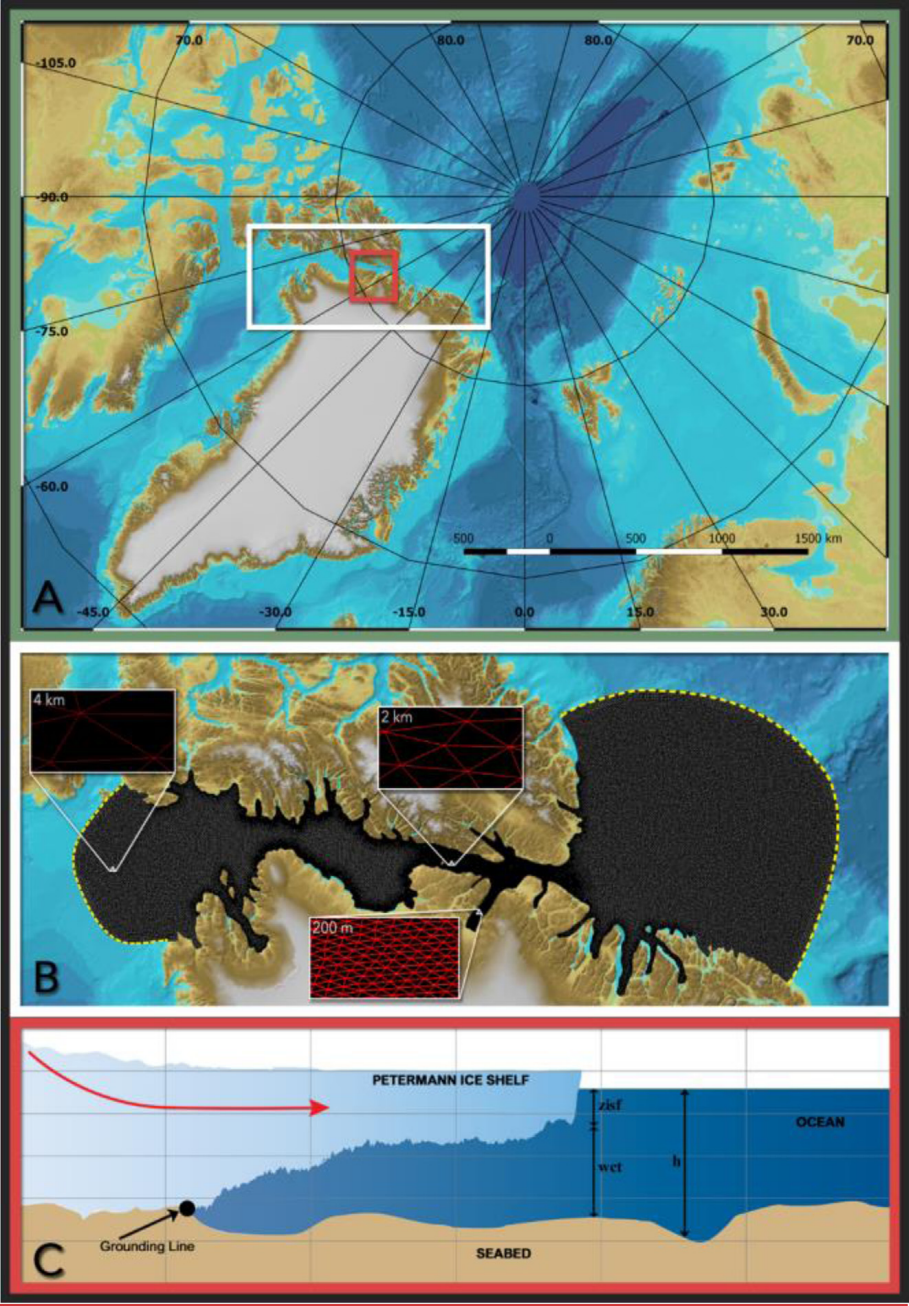
(A) Overview map (modified from IBCAO v3.0, [17]): The extent of the modeling domain is represented by a white box. The box in red indicates the location of the Petermann Glacier Ice Shelf (PGIS) and fjord within the Greenland Ice Sheet (GrIS). Longitude and latitude are expressed in °east and °north, respectively. (B) Zoom into panel (A), showing the unstructured FVCOM mesh with three distinct horizontal resolutions of 0.2 km inside the Petermann Fjord, 2 km in the neighbouring areas outside the fjord and 4 km elsewhere. The northern (Lincoln Sea) and southern (Baffin Bay) open boundary regions are highlighted in yellow. (C) A cross-sectional conceptual illustration of the Petermann ice shelf and ocean configuration: The red arrow indicates the direction of PGIS flow and the open ocean. The grounding line (GL) is the boundary across which the grounded glacier becomes afloat. Under the ice shelf, the water column thickness (WCT) is defined as the distance from the seabed to the base of the ice shelf, otherwise, it is the distance from the seabed to the sea surface. The depth of the seafloor (h) and the PGIS draft (zisf) are also indicated.

The recent surge in mass loss rates from the Greenland Ice Sheet (GrIS) is largely driven by an increase in ocean heat flux at the marine-ice margins [23,44]. Petermann, a large marine-terminating outlet glacier which drains the Northwest sector of GrIS [Fig. 1(A) and (B)], loses ∼80% of its mass across its ∼20 km wide grounding line (GL) [Fig. 1(C)] via channelized basal melting of its floating ice shelf [36]. The Petermann Glacier Ice Shelf (PGIS) draft is ∼600 m below sea level at the GL, gradually thinning to about ∼150 m near the calving front over a length of ∼50 km [[31], [37]]. Bathymetric profiles underneath the PGIS revealed a water cavity that is several hundreds of meters thick beneath the deepest regions of the ice shelf, and a 540–610 m deep inner sill roughly 25 km from the GL [45]. PGIS terminates in the ∼90 km long and ∼20 km wide Petermann Fjord, which opens up at Hall Basin in the Nares Strait. The Nares Strait is a long (∼500 km) and relatively narrow (∼30–50 km) waterway that connects the Lincoln Sea and the Baffin Bay, serving as an important pathway for heat and freshwater supply between the Arctic Ocean and the western subpolar North Atlantic Ocean [25]. Warm and salty waters of Atlantic origin in the Lincoln Sea have been shown to make their way downstream into the Petermann Fjord via the Nares Strait [22,46]. Geophysical mapping of the fjord bathymetry revealed considerable small-scale lateral variations and a 350–443 m deep outer sill at the fjord entrance [18,22]. Furthermore, the PGIS has been reported to have small-scale basal channels [[31], [36]]. These small-scale topographic features are suggested to impose a strong control on the circulation and meltrates at PGIS [36,45]. To investigate the formation of basal channels at PGIS, Gladish et al. [11] set up an idealized two-dimensional (2-D) coupled numerical ice shelf-ocean plume model. Their study demonstrated that these channelized sub ice shelf features are formed as a result of basal melting and help to increase the stability of the ice shelves. Millgate et al. [28] used a three-dimensional (3-D) configuration of the Massachusetts Institute of Technology general circulation model (MITgcm) with an idealized PGIS and fjord geometry to show the influence of basal channels on the cavity circulation and ocean induced melting of the ice shelf, thereby explaining how these features help to increase the stability of ice shelves. Furthermore, modeling studies conducted for another Greenland glacier by Xu et al. [[49], [50]] using a 2-D and a high-resolution 3-D configuration of the MITgcm revealed melt rate sensitivity of Store Glacier, western Greenland to the ocean thermal forcing and the subglacial discharge flux. Similarly, at Petermann, Cai et al. [3] used a 2-D MITgcm setup which incorporated the observed along fjord bathymetry from Tinto et al. [45] and the ice shelf geometry from Operation Ice Bridge, to model the basal melt rate sensitivity to ocean thermal forcing and subglacial discharge flux. Most noticeably, they showed that compared to winter, enhanced subglacial discharge during the summer period results in a two-fold increase in the maximum basal melt rates. Shroyer et al. [41] used the MITgcm to setup a regional model centered on the PGIS and fjord to study the response of PGIS to seasonal changes in the Nares Strait sea ice cover, however, their study used a smooth ice shelf that did not resolve the basal channels and did not include subglacial discharge. They found that sea ice transitioning to a mobile state in the summer induced a change in the ocean circulation in the Nares Strait which enhanced basal melting beneath the outer portion of PGIS by ∼20%. Therefore, a wide range of physical processes modulated by the local small-scale topographic features act to enhance or impede the heat transfer to the ice-shelf cavity by the ocean. Numerical modeling of basal melting in such highly dynamic composite environments characterized by complex geometry requires sufficient representation of the far field variability, as well as the capability to effectively resolve the small-scale local processes in the fjord and at the ice shelf-ocean interface.

For this study, we chose the Finite-Volume Community Ocean Model (FVCOM) [4,5] which uses an unstructured grid, a free-surface, and the 3-D primitive equations. The Finite-Volume numerical approach combines the flexible mesh topology of Finite Element Method and the simplistic discrete coding structures of the Finite Difference Method. The discretized integral form of the governing primitive equations of ocean circulation are numerically solved by evaluating fluxes over an unstructured triangular grid which provides an accurate numerical representation of local and global mass, momentum, heat and salt conservation. The computational efficiency, capability of accurately fitting complex irregular coastal boundary and topographic geometry and an accurate representation of the conservative laws in such environments make FVCOM ideal for our study region.

The computational grid in the horizontal direction is comprised of non-overlapping unstructured triangular cells, with 3 nodes, a centroid and 3 sides making up each unstructured triangle. The scalar variables such as temperature, salinity and sea-surface elevation are placed at the nodes whereas velocities u and v are placed at the centroids. In our setup, the irregular bottom and the PGIS draft are represented using terrain following sigma coordinates and the governing equations are solved in spherical coordinates under the hydrostatic approximation. An ice shelf module has been implemented into the FVCOM version [51] used in this setup which allows us to prescribe realistic Petermann Glacier Ice Shelf (PGIS) geometry for studying the ice shelf-ocean interactions. Further, to represent the effect of sea ice cover at high latitudes in modulating the atmospheric forcing on the ocean surface, a new sea ice module (Ice Nudge) has been implemented into the version of FVCOM used in this setup that allows to prescribe sea ice conditions as external surface-forcing parameters.

Setting up the model requires an unstructured triangular grid, bathymetry and ice shelf draft, initial and lateral ocean boundary conditions, and sea ice and atmospheric forcing. The following sections describe procedures to systematically set up these specific components in detail. The performance of our first simulations using these components are discussed and validated against conductivity-temperature-depth (CTD) profiles that provide information on the vertical structure of temperature and salinity from the Petermann Fjord taken during the 2019 Ryder expedition [19].

## The FVCOM grid and the modeling domain

The model grid was generated using Distmesh [35]. This program provides an efficient method for generating unstructured and triangular meshes of high quality (i.e., with triangles being close to equilateral) and is available as Matlab code. It applies a simple algorithm that combines the physical principle of force equilibrium in a 2-D truss structure (a structure of springs) with a mathematical representation of the geometry using Signed Distance Functions (SDFs). Given the coordinates of a point in space, the function returns the shortest distance between that point and the boundary of the domain. The sign depends on the point lying inside (negative) or outside (positive) the domain. Distmesh creates an initial distribution of equilateral triangles within a bounding box, from which a set of nodes interior to the computational domain are extracted based on the SDFs. Then, re-triangulation is done using the Delaunay algorithm and the nodes are moved iteratively to obtain an equilibrium configuration by minimizing the potential energy of the truss structure. Finally, a termination criterion is applied when all the nodes are fixed in space.

In the vertical direction, the bottom boundary of the modeling domain is given by the bathymetry of the ocean/fjord floor. The upper boundary is given by the draft of the floating Petermann Glacier tongue, and the height of the free surface seaward of its calving front. All heights are expressed relative to the mean sea level using the geoid eigen-6c4 [9]. Lateral boundaries are given by the coastline where applicable, and is comprised of open ocean boundaries otherwise. The unstructured triangular grid features a total of 112,709 nodes including 309 open boundary nodes that are distributed along the northern and southern boundary [Fig. 1(B)] and 23 terrain-following sigma layers in the vertical.

Our regional modeling domain is located between 75**°**N and 87**°**N and between 29**°**W and 81**°**W. The domain is designed to mainly cover the Nares Strait and the Petermann Fjord, and is divided into several polygons with different horizontal resolution [Fig. 1(A) and (B)]. This allows us to balance the need for computational efficiency with needs for sufficiently detailed representation of, e.g., coastline, bathymetry and its changes over small spatial scales, and small-scale circulation processes in the cavity beneath the Petermann Glacier's floating tongue. Inside the Petermann Fjord, the horizontal grid resolution is 0.2 km, sufficient to mediate steep seafloor and ice shelf basal topography errors [Fig. 1(B)]. Outside the fjord, the resolution is relaxed to 2 km for parts of the Nares Strait and is ultimately set to 4 km near the open boundary regions [Fig. 1(B)]. Two open boundary regions to the north and south [Fig. 1(B)] are set to allow for investigations of the impacts of the warm and saline Atlantic inflow from the Lincoln Sea (north) and its extension southward into Baffin Bay, to the basal melting of PGIS. The coastline bounding the modeling domain was retrieved from the GSHHG (Global Self-consistent, Hierarchical, High-resolution Geography) database [47] and corrected for the location of the front of the Petermann Glacier. All coastline points were smoothed and redistributed in an averaged interval of 0.2 km.

## Setup of the model bathymetry and ice shelf topography

In our setup, the water column thickness (WCT) for regions of the Petermann ice shelf is defined as the distance from the seabed to the ice shelf base [Fig. 1(C)], and otherwise it is the distance from the seabed to the ocean surface. Bathymetry in the Petermann Fjord and ice-draft geometry for the Petermann Glacier are derived from the BedMachine Greenland v3 bed topography and ice thickness dataset [29], provided at a spatial resolution of 150 m. However, underneath the ice shelf, the bathymetry is poorly constrained in this dataset, and therefore, additional changes based on the IceBridge aerogravity data [45] and manual corrections have been implemented as described below. Outside the Petermann Fjord, 1-km grid bathymetry data from IBCAO v3.0 [17] is used.

### Bathymetry smoothing

A sigma layer (terrain-following) vertical coordinate system is used in FVCOM to capture the irregular bottom topography and the PGIS draft (for details concerning the transformation of the governing equations to the σ-coordinate system, see [4]), which, however, still needs to be smoothed relative to the grid scale to minimize errors in the discretization of horizontal pressure gradients along the sloping σ-layers. Smoothing is achieved by requesting that the slope factor, namely the maximum of r (h, e, e') = |h(e) - h(e')| / [h(e) + h*(*e')] over any two adjacent wet cells e and e' in the σ-coordinate ocean model, with corresponding bathymetries h(e) and h(e'), respectively, satisfies *r* ≤ 0.2 (note that 0 ≤ *r* < 1), see [[2], [42]].

The BedMachine v3 dataset was gridded and smoothed (*r* ≤ 0.2) to the 0.2 km resolution grid points (see below). Also, the IBCAO v3 bathymetry dataset was gridded and smoothed using a mean filter to the 2 and 4 km resolution grid points, such that *r* ≤ 0.2. Then, individual weight coefficient matrices (WCM) at each grid resolution were defined as:

• WCM 0.2km: changes linearly from 1 to 0 as the resolution changes from 0.2 km to 2 km, elsewhere, it is set to 0.

• WCM 2km: changes linearly from 1 to 0 as the resolution changes from 2 km to 4 km or 2 km to 0.2 km, elsewhere, it is set to 0.

• WCM 4km: changes linearly from 1 to 0 as the resolution changes from 4 to 2 km, elsewhere, is set to 0.

Further, the smoothed bathymetry and the WCMs at each resolution are interpolated over the entire modeling domain. The weighted new bathymetry at each resolution is generated by multiplying the interpolated bathymetry matrix with the corresponding WCM at that resolution, yielding three distinct bathymetry matrices at the given resolutions

(1) $$H_{0.2}=h_{0.2}\times \mathrm{WC}M_{0.2,}H_{2}=h_{2}\times \mathrm{WC}M_{2,}H_{4}=h_{4}\times \mathrm{WC}M_{4}$$

where, h_0.2,_ h_2,_ and h_4_ are the interpolated smoothed bathymetries on the FVCOM nodes and WCM_0.2_, WCM_2_ and WCM_4_ are the interpolated WCMs at 0.2 km, 2 km and 4 km resolution. H_0.2_, H_2_ and H_4_ are the resulting weighted bathymetries at 0.2 km, 2 km and 4 km resolution. Finally, the weighted bathymetry matrices are superposed, ensuring that bathymetric variation (steepness) is captured in sufficient detail for all grid resolutions employed

(2) $$H=H_{0.2}+H_{2}+H_{4}$$

where, H is the final model bathymetry to be used in simulations.

### Adjustment and smoothing of the Petermann Fjord bathymetry

#### Modifying the BedMachine v3 dataset

**Fig. 2:**
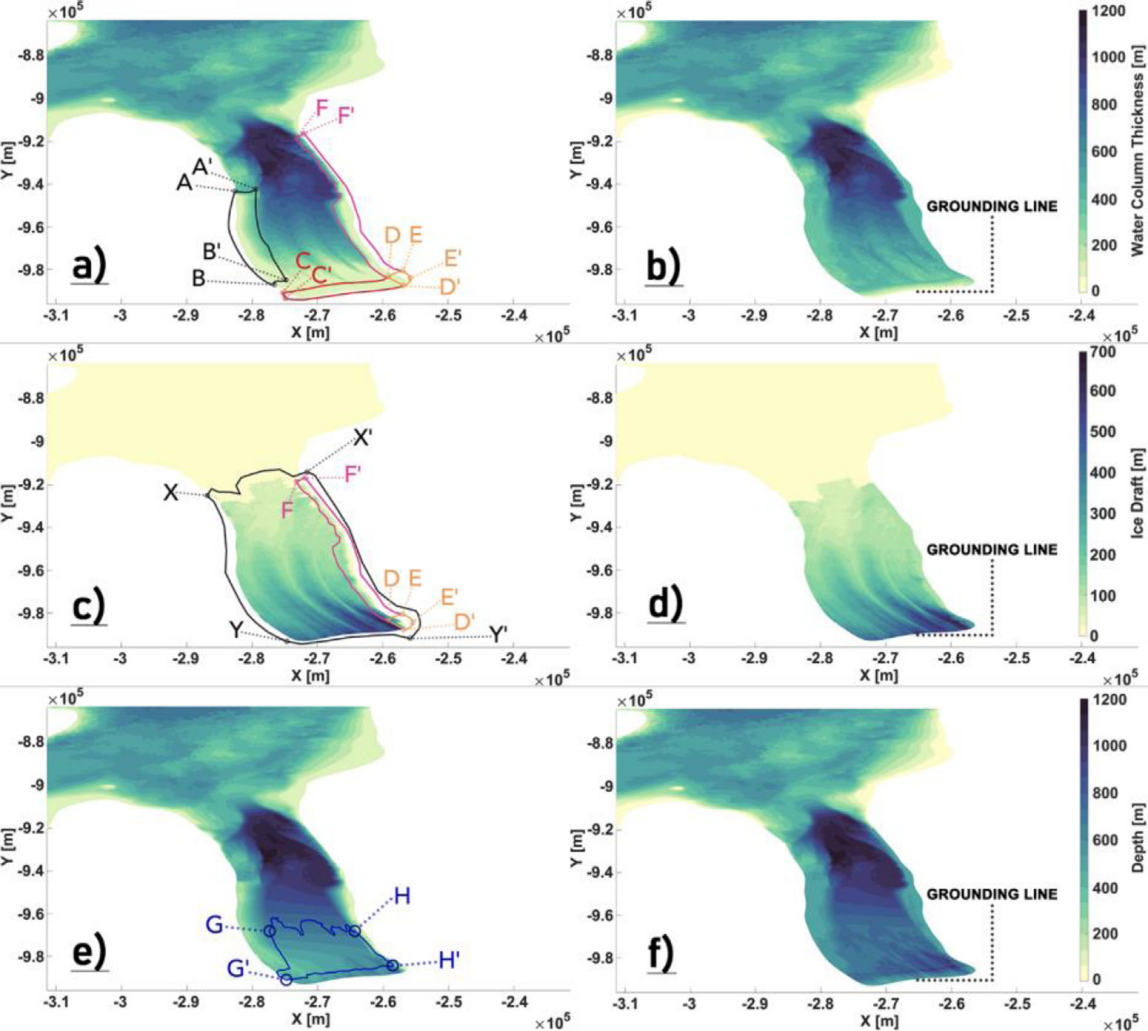
Original (a, c, e) and locally modified (b, d, f) BedMachine data in the Petermann Fjord. Easting and northing on x- and y- axis, respectively. (a,b): Water column thickness (WCT), (c,d): Ice-Draft (zisf), (e,f): Bathymetry (h).

The BedMachine v3 Petermann Fjord bathymetry for areas north of the 2015 calving front comes from the multibeam bathymetric mapping that was carried out during the Petermann 2015 Expedition [18]. In the absence of seafloor depth measurements underneath the ice shelf, the bathymetry is derived by extending the multibeam data southward to match the ice draft depth at the GL using a natural neighbor interpolation. The sub ice shelf bathymetry determined using reflection seismics at other Greenland and Antarctic glaciers [14,^26^,27] have revealed several hundreds of meters of WCT beneath the deepest parts of the ice draft. Therefore, such extended areas of thin/negligible WCT near the GL derived from the BedMachine v3 dataset [Fig. 2(a)] is not a likely representation of the actual sub ice shelf topography at Petermann. Several regions of interest underneath and around the floating ice tongue were identified, for which modifications to the bathymetry (h), ice-draft (zisf) and the WCT are suggested, in order to provide a physically plausible and numerically robust (in regard to pressure gradient errors) geometry. These are explained and defined below:

• *Western Flank*: Along the western flank defined by the polygon AA'BB' [Fig. 2(a)], we see a sharp transition in the WCT from ∼400 to ∼0–50 m along the north to south profile, and we observe a similar transition from east to west over a small horizontal distance. Here, gradients in WCT are provided to ensure that it is smoothly extended from north to south and from east to west [Fig. 2(b)].
• *Eastern flank:* There is a marginal coastline mismatch between the model and the dataset. The Petermann Fjord coastline in the model extends further east into regions where the WCT and ice-draft are negligibly small or non-existent (fjord wall), resulting in steep gradients in WCT and zisf. A simple method to account for such an inconsistency is to classify all nodes within the polygons DEFF' and DD'EE' [Fig. 2(a) and (c)] as wet (sea) nodes instead of dry (land) nodes by extending the dataset eastward to match the model coastline. As such, along the entire right flank defined by the polygon DEFF', we enforce a minimum ice thickness of 100 m [Fig. 2(c) and (d)] and ensure that the WCT is 400 m [Fig. 2(a) and (b)]. In the polygon region defined by DD'EE' [Fig. 2(c)], we argue that similar to the ambient ice shelf thickness [in polygons DEFF' and CC'DD' in Fig. 2(c)], the ice-draft close to the GL is also deeper than the dataset suggests. Therefore, the ice thickness in this region is set close to the ambient ice shelf thickness [Fig. 2(d)]. Furthermore, to ensure a smooth transition to negligible WCT near the GL, we define a linear gradient in WCT that decreases from 350 to 50 m close to the GL [(Fig. 2(a) and (b)].
• *Central Zone*: The ice-draft is deepest close to the GL, and is gradually becoming shallower towards the fjord mouth. Compared to the thickness of the ice in regions defined by a deep (> 250 m) draft, the bathymetry is too shallow [Fig. 2(c) and (e)], which renders the WCT negligible (∼50–100 m). As argued above, based on the reflection seismic studies conducted at other Greenland and Antarctic glaciers, it is reasonable to assume that the bathymetry in these regions is deeper than suggested by the dataset; and that there is a smoother transition towards a negligible WCT as the GL is approached. To ensure this, we first define a number of small polygons within the area enclosed by polygon GG'HH' [Fig. 2(e)], over which the bathymetry is deepened [Fig. 2(f)], the extent of which is governed by the depth of the draft [Fig. 2(c)]. Thereafter, to enforce a smooth transition, we define another polygon region CC'DD' [Fig. 2(a)] and set a linearly decreasing WCT which ranges from 400 to 50 m close to the GL [Fig. 2(b)].
• We define a polygon region XX'YY' enclosing the Petermann ice shelf [Fig. 2(c)] and remove all ice outside this region. Coastline mismatch in the PGIS region can create patches of negligible WCT and zisf or dry nodes. While most such regions along the eastern flank were identified and fixed as outlined above, it is safe to assume that several nodes within the PGIS domain, particularly along the western flank and the calving front, could still remain unidentified. Likewise, small islands outside the PGIS and fjord that are not identified in the model grid can also result in dry nodes. To avoid any steep gradients in WCT resulting from such discrepancies, we ensure a minimum ice draft depth of 30 m for regions inside XX'YY' with 0<zisf<30, and a minimum WCT of 50 m throughout our modeling domain.

#### Implementing the inner sill

The bathymetry beneath the Petermann ice shelf was modelled along a few selected 1D survey tracks by Tinto et al. [45] using the aerogravity data from Operation Ice Bridge (IB) in conjunction with airborne lidar and radar data and ship based bathymetry soundings. An inner sill at 540–610 m depth was revealed about 25 km from the GL resulting in a minimum water column thickness of 400 m. A total of six survey tracks were flown along the axis of the fjord. One of the lines was flown at a higher elevation and the gravity data from it were too smoothed out to be inverted. Therefore, the IB bathymetry models from the remaining 5 lines are used in this study.

**Fig. 3:**
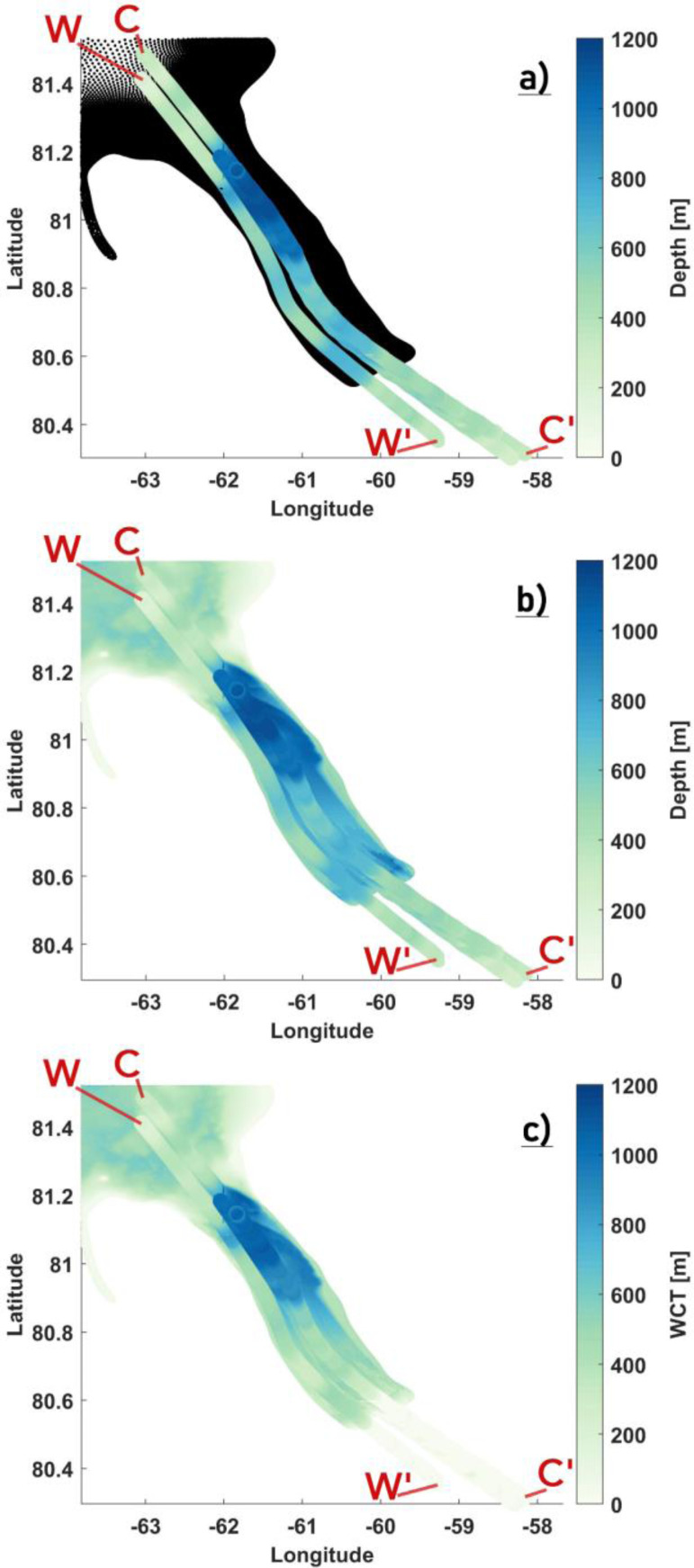
Bathymetry revealed by the IB survey tracks plotted over (a) the model nodes and (b) the locally modified BedMachine bathymetry [from Fig 2.(f)]. (c) Derived water column thickness (WCT) from the IB survey tracks and the locally modified BedMachine WCT [from Fig 2.(b)] are shown. WW’ and CC’ denote the western and central IB tracks. Longitude and latitude are expressed in °east and °north, respectively.

**Fig. 4:**
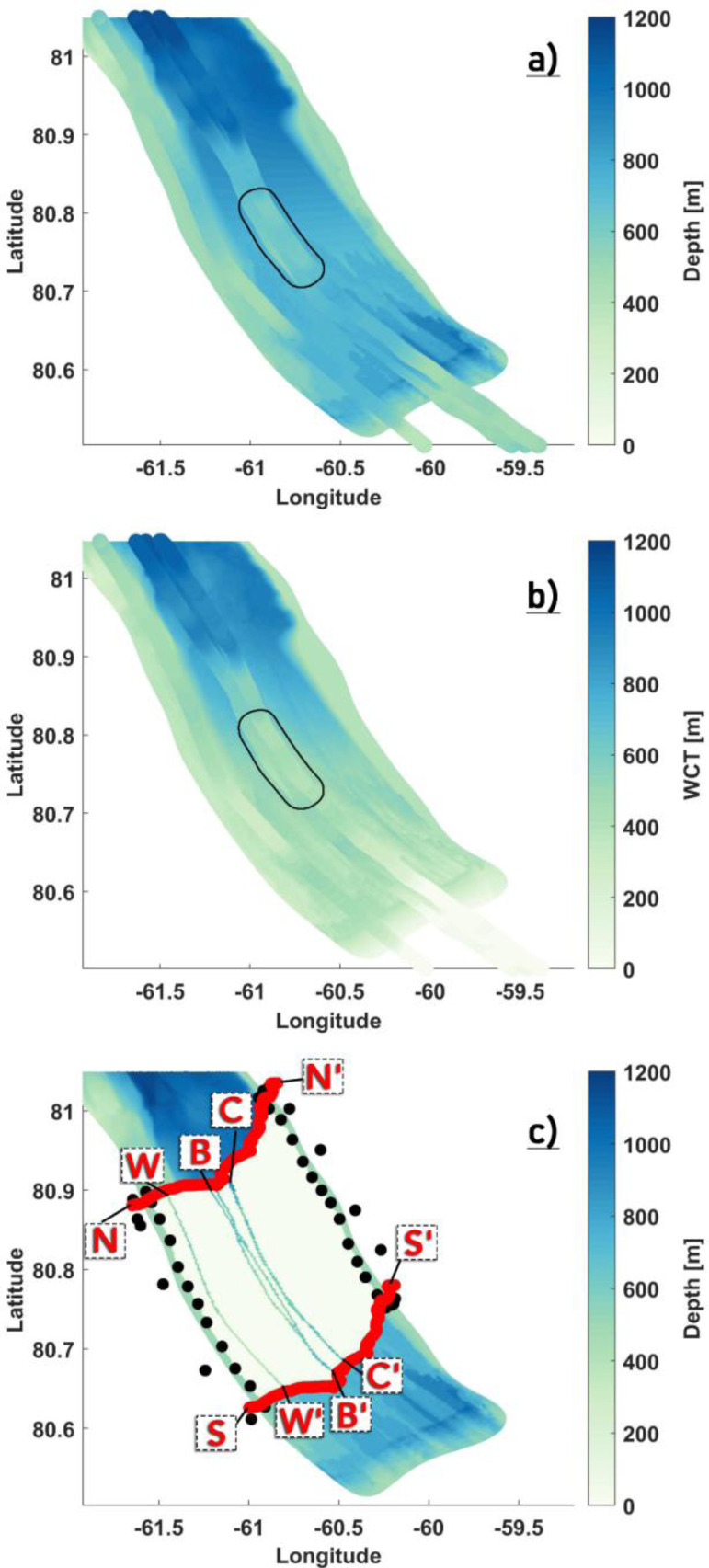
Bathymetry (a) and derived WCT (b) from the IB survey tracks plotted over the locally modified BedMachine bathymetry and WCT. Location of the inner sill ∼ 25 km from the GL (a,b) revealed by the IB survey tracks is highlighted. (c) The north-south extent of the ROI polygon to make modifications around the inner sill is denoted by NN’ and SS’, respectively, with the padded zones on the eastern and western flanks demarcated using black circles. (c) WW’ and CC’ denote the western and central IB tracks with BB’ in between. Longitude and latitude are expressed in °east and °north, respectively.

**Fig. 5:**
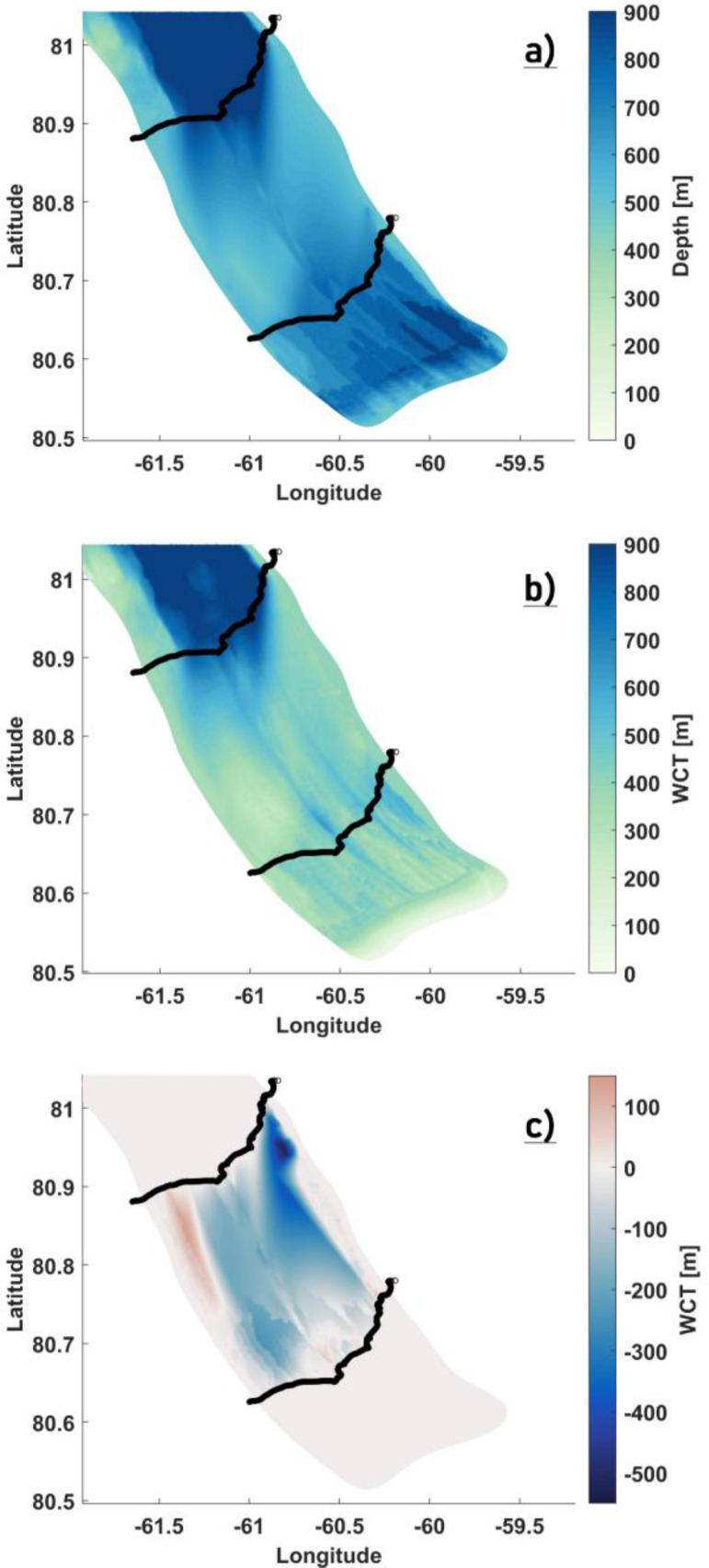
The locally modified bathymetry (a) and WCT (b) maps showing how the inner sill is represented in our model. The north-south extent of the ROI polygon to make modifications around the inner sill are highlighted in black. Note: The colorbar in (a) and (b) are exaggerated to highlight the inner sill. (c) WCT difference [(b) - Fig. 2(b)] map. Longitude and latitude are expressed in °east and °north. respectively.

The distribution of the survey tracks across the fjord is shown in Fig. 3(a). Tracks CC’ and WW’ surveyed the central and western side of the fjord. The remaining three lines are all clustered around CC’ with a maximum separation of 2.5 kms, with one of them repeating the CC’ track to within 100 m lateral separation. With a maximum width of ∼ 17 km, the Petermann Fjord is wide and the east-west coverage from the IB tracks is sparse. Furthermore, there is no information regarding the eastern half of the fjord. As such, preparing a realistic bathymetry map for the entire fjord [as shown in Fig. 2(f) above] using only the survey tracks is not possible. A comparison is made between our topography maps [from Fig. 2(b) and (f)] and the IB bathymetry models and adjustments are made only where it is necessary. To that end, we notice that our modified maps [from Fig. 2(b) and (f)] largely agree with the IB bathymetry models and provide a fairly accurate representation of the cavity geometry [Fig. 3(b) and (c)]. Major discrepancies between our maps and the IB bathymetry models, aside from a dynamically important inner sill [45] are close to the GL, where our bathymetry around the central track CC’ appears to be deeper than the IB modelled depths [Fig. 4(a)]. As argued above (for modifying the central zone), deepening was done to ensure a smooth transition towards a negligible WCT, which in principle is what is seen in the derived IB WCT map [Fig. 4(b)]. Also, there is considerable lateral variability in this region [Fig. 4(a) and (b)] between the survey tracks, with IB modelled depths of closely separated tracks being deeper and more in agreement with our values. Therefore, we argue against making further modifications to our topography maps in this region without any additional information. Moving over to the inner sill region, sparse coverage from the IB tracks (as stated above) implies that the across fjord variation of this sill is largely unknown, particularly towards the eastern half of the fjord. Therefore, to extend the sill laterally and to provide a full 2D depth field beneath the ice shelf, certain necessary assumptions are made and the following steps are taken to prepare the final maps:

• *Defining a region of interest (ROI) polygon around the inner sill*: Heading glacierward along the survey tracks [Fig. 4(a)], the BedMachine bathymetry is seen to depart from the IB modelled depth values ∼ 5 km north of the inner sill. This being the sub ice shelf cavity region, the IB modelled depth values are more likely to provide an accurate picture and are therefore used instead of the BedMachine bathymetry in our final map. Here, a cross-section NN’ [Fig. 4(c)] is used to mark the northern boundary of the polygon. Continuing southward, the inner sill is encountered and thereafter, the modified region defined by the polygon in Fig. 2(e). There is strong agreement between our modified values within this polygon and the IB modelled depth values [Fig. 4(a)]. Therefore, the southward extent of the inner sill marks the southern boundary of the polygon [Section SS’ in Fig. 4(c)]. Bathymetry at all nodes inside the bounded box defined by NN’ and SS’ is set to zero.
• *Assumption*: The eastern and western flanks are padded with pre-existing depth values [from Fig. 2(f)] to ensure smooth transitions to shallower depths as the coastlines are approached [Fig. 4(c)]. Assuming little to no across fjord variation in the inner sill depth, the IB survey tracks are replicated across the width of the fjord [Fig. 4(c)]. To that end, tracks CC’ and WW’ are used to extend the sill towards the eastern and western coastline, respectively, [Fig. 4(c)]. Likewise, track BB’ is replicated to populate the region bounded between BB’ and WW’ [Fig. 4(c)].
• *Interpolation*: IB modelled depth values from the replicated tracks are used to interpolate to all the nodes inside the ROI polygon, where natural neighbor interpolation is used to create a smooth final product [Fig. 5].

## Initial and boundary conditions

**Fig. 6:**
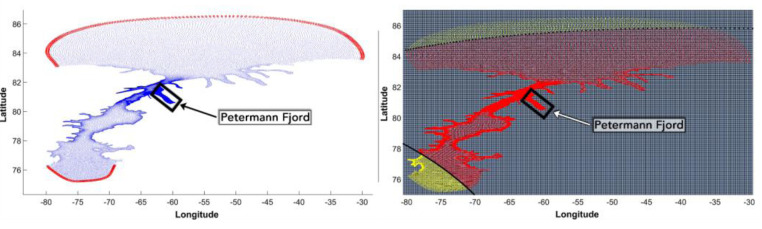
(a) Distribution of the FVCOM nesting nodes (in red circles) along the northern and southern boundary of the modeling domain. A total of 618 nodes are identified using a nesting zone radius of 5 km. (b) The atmospheric forcing setup for FVCOM. The outline of the 5.5 km high-resolution RACMO domain is highlighted by black circles. The FVCOM nodes that lie inside it are shown in red and those falling outside are shown in yellow. The ERA-Interim grid covering the entire FVCOM domain is shown in blue. Longitude and latitude are expressed in °east and °north, respectively. The black box in (a) and (b) shows the location of the Petermann Fjord.

At the start of a simulation, FVCOM requires initial conditions for the prognostic state variables of temperature, salinity, velocity and dynamic sea surface height anomalies at all nodes/cells of the domain. Further, time series of boundary conditions for these variables need to be prescribed at the open boundary nodes/cells (see below) and at the forcing time interval chosen for the simulation [Fig. 6(a)].

**Fig. 7:**
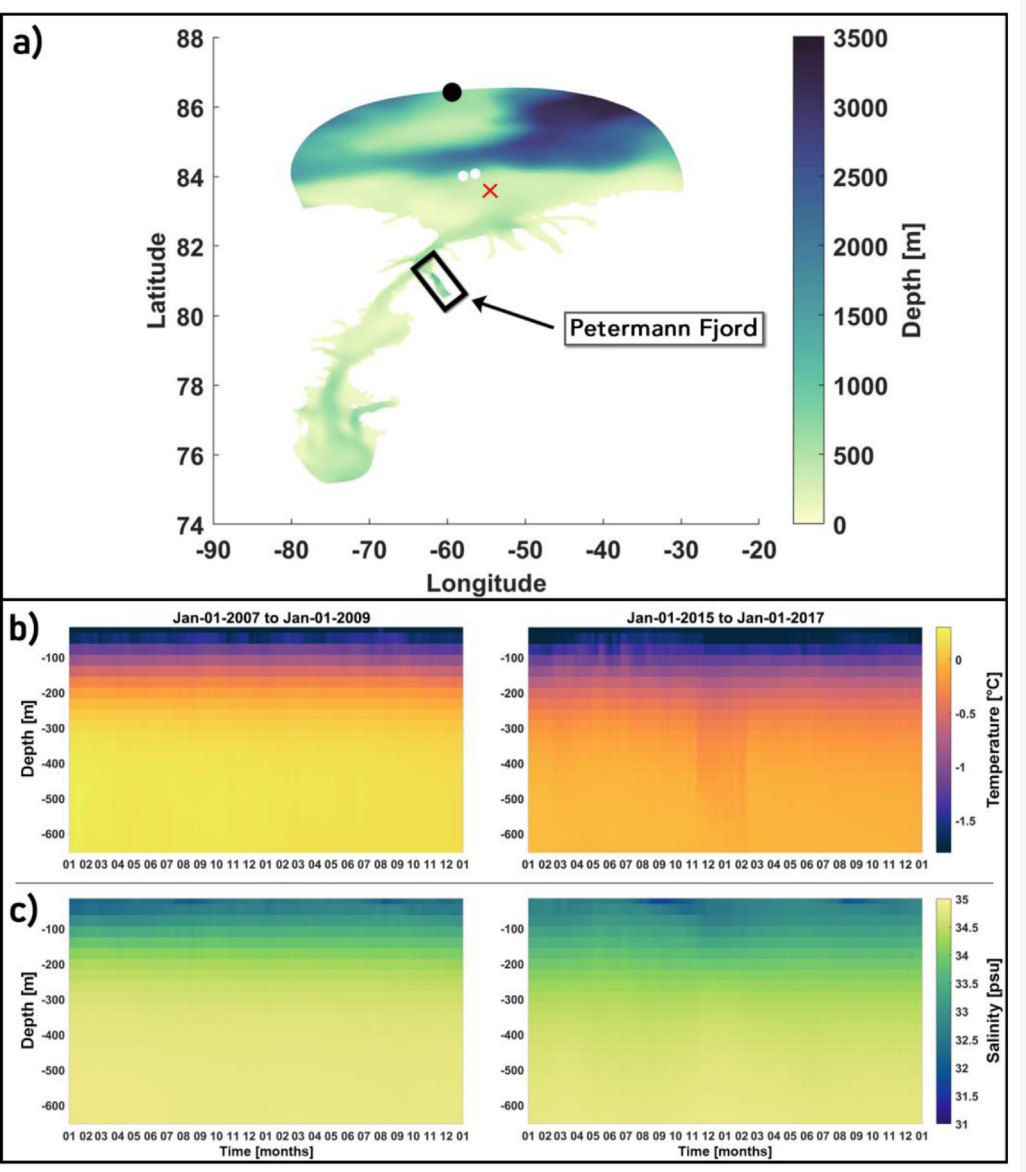
(a) Black circle showing the location of the northern boundary node (overlaid over the model bathymetry map) at which the daily averaged A4 temperature (b) and salinity (c) time-series are shown for two periods. The northern sill region is highlighted by a red ‘X’ symbol and approximate locations of the moorings deployed by Münchow et al. [30] are shown as white circles. Longitude and latitude are expressed in °east and °north, respectively. The black box shows the location of the Petermann Fjord.

Initial velocities at the cells are set to zero, and initial conditions for salinity and temperature at the nodes are obtained from the structured grid Regional Ocean Modelling Software (ROMS) model [39], operating on a pan-Arctic domain with a 4 km horizontal resolution (hereafter referred to as A4) in a simulation covering the years 2007–2017 (for details regarding the A4 setup and forcing, see [12]). The A4 output for July 1, 2014, 00:00:00 h (UTC) is extracted to initialize FVCOM, following which, it is to run until January 01, 2017 00:00:00 h (UTC), nested into the A4 sea surface elevation, temperature, salinity and velocity fields at the open boundaries (see below). These fields are updated hourly, where we have chosen the interval to match the hourly sampling of the sea surface elevation field from the Arctic Ocean Tidal Inverse Model (AOTIM) [33] which is to be added to the A4 sea surface elevation field, as explained below. However, upstream in the Arctic basin, there is a cold bias in the A4 Atlantic water (AW) temperatures that persists throughout the run which affects nudging at the northern boundary [Fig. 7(a)–(c)]. To alleviate this issue, monthly climatologies of temperature, salinity and velocity are constructed using a period from the start of the A4 run (2007–2009) when the bias is negligible, which are then interpolated on to the hourly forcing intervals. The modelled A4 AW temperature of ∼ 0.22 °C and salinity of ∼ 34.84 psu obtained from this period (2007–2009) are relatively closer to the reported values [30] when compared to the 2015–2017 period [Fig. 7(b) and (c)] and provide a more controlled far-field forcing. Note that our northern boundary location lies further north of the moorings that were deployed by Münchow et al. [30] off the shelf slope, north of the northern sill in the Lincoln Sea [Fig. 7(a)]. Here, off the shelf slope, at depths of ∼490 m, they found an AW signature of θ ∼ 0.4 °C and S ∼34.86 psu, where θ is potential temperature and S is salinity. However, these types of AW have been found to occupy the 200–500 m depth and can have temperatures ranging from 0.28 °C ≤ θ ≤ 0.8 °C [46].

In ROMS, the variables of sea-surface elevation, temperature and salinity are placed at the center of the grid cell (ρ-type variable) and the horizontal velocity components u,v are placed at the east-west and north-south edges of the grid cell (u and v-type variable) respectively [39]. To achieve the nesting, the ρ, u and v point indices of the structured grid bordering the FVCOM grid are obtained, which are used to crop the pan-Arctic A4 grid around the FVCOM grid. The ocean ρ point indices (ρ_ocn_) are selected such that

(3) $$\rho_{ocn}=\rho \left(\rho_{m}==1\&u_{m}==1\&v_{m}==1\right)$$

where, ρ_m_, u_m_ and v_m_ are the land-sea masks (0 for land, 1 for sea) on ρ, u and v points, respectively. Symbols ‘==’ and ‘&’ are, respectively, the relational and logical operator. ρ_ocn_ is then used to obtain the A4 grid information at the ocean-only ρ points.

For each FVCOM node and cell center, the four nearest (Euclidean distance) A4 ρ_ocn_ points are identified, and their bilinear interpolation coefficients (α) and indices are computed. In case there are less than four A4 ρ_ocn_ points available, indicating that the FVCOM node lies close to land, the nearest A4 ρ_ocn_ point is chosen as the index. The nearest four (node) coefficients and indices are used to interpolate the A4 topography to the FVCOM node points in the nesting zone. As such, by forcing the FVCOM bathymetry to be the same as that of the larger model at the nesting zone, we ensure that the volume is conserved when FVCOM is nested into other larger models. To do so, the minimum of the Euclidean distance (R) between the FVCOM boundary nodes and each of the 112,709 horizontal node points (x_n_, y_n_) is computed. A nesting zone radius (R_nest_) of 5 km, which is the distance from the FVCOM boundary, is defined. Two radii *r*_1_ = 1.1 x R_nest_ and *r*_2_ = 2 x R_nest_ are used to generate a weight function matrix as follows:

For *n* = 1: number of nodes (here, 112,709)

If R (x_n_,y_n_) < r_1_, a weight ω(x_n_,y_n_) of 1 is assigned

If R (x_n_,y_n_) > r_2_, a weight ω(x_n_,y_n_) of 0 is assigned, and,

If r_1_ < R*(*x_n_,y_n_) < r_2_, then

(4) $$a=\frac{1}{r_{1}-r_{2}};b=\frac{r_{2}}{r_{2}-r_{1}},$$

(5) $$\omega_{\left(x_{n},y_{n}\right)}=a\times R\left(x_{n},y_{n}\right)+b$$

The interpolated topography h_interp_ is then calculated as

(6) $$h_{\text{interp}}=h_{0}\times \omega +h_{fv}\;\times \left(1-\omega \right),$$

where h_fv_ is the FVCOM topography and

(7) $$h_{o\left(n\right)}=\sum_{j=1}^{4}\alpha_{\left(n,j\right)\times }h_{\left(n,j\right),}$$

where α_n,j_ denotes the nearest four [*j* = 1:4] coefficients and h_n,j_ is the A4 topography extracted at the nearest four [*j* = 1:4] indices surrounding the node ‘n’, for *n* = 1 ∶ number of nodes (here, 112,709). All nodes/cells within the radius R_nest_ lie in the nesting zone and are used to extract the indices of the nesting grid cells, and subsequently, the nodes. The nesting grid is then generated, containing the indices and coordinates of the nesting nodes and cells, the topography at the nesting nodes and node identity of the three nodes that make up a cell for each of the nesting cells. In the next step, the vertical interpolation coefficients and indices for each of the FVCOM node and cell center are obtained. Here, the previously determined coefficients and indices for horizontal interpolation are used to interpolate the ROMS layer depth at ρ-points to the FVCOM nodes and cell centers. Then, at each of the 23 FVCOM (vertical) σ-levels, the nearest depth points from A4 are identified and the corresponding vertical indices and coefficients are computed. Lastly, using the cropped indices, A4 ocean ρ grid, the horizontal and vertical interpolation coefficients and indices and the nesting grid, the required nesting variables from A4 are interpolated onto the FVCOM nodes and cells over all the simulation time steps.

The AOTIM, an 8-constituent regional inverse tide model of the entire Arctic Ocean is used to obtain the phases and amplitudes of the tidal waves at the FVCOM boundary. Padman and Erofeeva [33], show that for the Arctic Ocean, averaged over their entire model domain, the M2, S2, K1 and O1 tides are the four most energetic tidal constituents accounting for 95% of the total (based on 8-constituent) tidal potential energy (Table 1). The M2 tide dominates the tidal height variability in the Arctic with amplitudes exceeding 1 m in the southern Barents Sea near the entrance to the White Sea, in the Labrador Sea and at the northern margin of Baffin Bay. The amplitude distribution for S2 is similar to M2, though being about a factor of three smaller. K1 and O1 tides are largest in the Baffin Bay and the Labrador Sea and in the Gulf of Boothia with maximum amplitudes of 0.4 m and 0.2 m, respectively. Therefore, in this setup, to better interpret the contribution from the dominating frequencies, only the M2, S2, K1 and O1 tides are included. The TMD (Tide Model Driver) Matlab toolbox [34] is used to extract the tidal harmonic constants (amplitudes and phase lag) for sea surface height and to make surface elevation time series predictions.

**Table 1:** Properties of the 4 major tidal constituents used in this study. The potential energy for each constituent, expressed as percent of the 8-constituent total, is taken from [33].

Tidal Constituent	Name	Period (in hours)	Potential Energy (in%)
Semi - Diurnal			
M2	Principal Lunar	12.42	79
S2	Principal Solar	12	10
Diurnal			
K1	Lunisolar	23.93	5
O1	Principle Lunar	25.82	1

The daily-averaged sea surface elevation extracted from A4 at the FVCOM nesting zone boundary nodes are interpolated on to the same (hourly) sampling as the AOTIM tides and the two fields are added together to form one product that drives the hourly tidal forcing at the nesting nodes.

## Surface forcing

### Atmospheric forcing

At the model surface, atmospheric variables are prescribed, namely 2-m air temperature, surface air pressure, relative humidity, eastward and northward wind speed, downward longwave radiation, net shortwave radiation, precipitation, and evaporation. These variables are derived from 3-hourly output from the polar (p) version of the RACMO2.3p2 (Regional Atmospheric Climate Model) covering the region north of 75° N for the period from 1958 to 2017 at a horizontal resolution of 5.5 km, which is important to resolve local meteorological conditions in the Petermann Fjord region. At its lateral boundaries, RACMO2.3p2 is forced by ERA-40 (1958–1978) and ERA-Interim (1979–2017) re-analyses on a 6-hourly basis within a 24–grid cell wide relaxation zone (see [32] for a description of the model). At gridpoints where the FVCOM domain exceeds the RACMO domain (along its northern and southern boundary), ERA-Interim (1979–2017) output is used to force FVCOM [Fig. 6(b)]. From the two atmospheric models, RACMO 2.3p2 and ERA-Interim (1979–2017), the obtained data are used to compute the bulk air-sea fluxes at the ocean surface. The bulk formula heat fluxes (sensible, latent and upward longwave radiation) and the net heat flux are calculated using the heat flux parameters which includes the 2-m air temperature, surface air-pressure, relative humidity, downward longwave and net shortwave radiation [[6], [7]]. The 10 m zonal and meridional wind speeds are used to compute the momentum fluxes. The difference between the rainfall and evaporation rate is used to compute the net surface freshwater flux. Note that these fluxes are only applied to the ice free fraction of a given cell area, whereas, for the ice covered fraction, these are provided by the Ice Nudge module (see below).

The ERA-Interim relative humidity field is calculated using the 2-m air temperature and dew-point temperature fields [1] as follows:

(8) $$e\;=6.112\;\times e^{\left(17.67\times T_{d}\right)/\left(T_{d}+243.5\right)\;},$$

(9) $$e_{s}=6.112\;\times e^{\left(17.67\times T\right)/\left(T+243.5\right)},$$

(10) $$RH=\frac{e}{e_{s}},$$

where e represents the vapor pressure in mb, e_s_ is the saturation vapor pressure in mb, T_d_ and T are the dew-point temperature and 2-m air temperature in °C, respectively, and RH is the relative humidity.

The ERA-Interim forecast fields of precipitation, evaporation and radiative fluxes are stored as accumulated fields, i.e., they are accumulated since the start of a forecast. The forecasts start at 00:00 and 12:00 UTC, and therefore with 3-hourly steps, these fields are accumulated twice each day over four 3-hourly periods, from 00:00 to 12:00 and from 12:00 to 00:00 UTC. As a result, precipitation and evaporation are stored in meter of water equivalent and the radiative fluxes are expressed in J/m^2^. To obtain values that are consistent with the instantaneous RACMO fields (in m/s and W/m^2^) that are valid at a given time-step, the accumulated fields were stored as follows:

In a semi-diurnal cycle, if t_0_ denotes the start of a forecast (at 00:00 or 12:00 UTC), then the first 3-hourly step after the start of the forecast (at 03:00 or 15:00 UTC) is designated as the t_0_+1 step such that

(11) $$\Phi_{e}\left(t_{0}+1\right)=\xi_{e}\left(t_{0}+1\right)\;/\;3\;\times \;60\;\times \;60\;s,$$

and at every other time step ($t_{0}+n$) within the semi-diurnal cycle, for n=2,3or4

(12) $$\Phi_{e}\left(t_{0}+n\right)=\left(\xi_{e}\left(t_{0}+n\right)-\;\xi_{e}\left(t_{0}+\left(n-1\right)\right)\right)/\;3\;\times \;60\;\times \;60\;s,$$

where Φ_e_ (t_0_ + 1) and Φ_e_ (t_0_ + *n*) represent the ERA-Interim field calculated at the 3-hourly time steps t_0_ + 1 and t_0_ + *n*, respectively. ξ_e_ (t_0_ + 1), ξ_e_ (t_0_ + *n*) and ξ_e_ (t_0_ + (n - 1)) are the accumulated values provided at t_0_ + 1, t_0_ + *n* and t_0_ + (n - 1), respectively. Note that Φ_e_ actually represents the average flux (or the mean of what is collected) between any two given time steps.

Lastly, all the instantaneous parameters from the ERA-Interim analysis fields which are provided at 6-hourly time steps are interpolated in time to match the 3-hourly RACMO forcing frequency.

The forcing period chosen here is consistent with the simulation period. To generate the forcing, the atmospheric grid is first cropped around the FVCOM grid and the corresponding longitude and latitude indices are stored. The interpolation coefficients and indices for the nearest four atmospheric grid points surrounding each of the FVCOM node and cell are then computed in the same manner as described in the previous section. Lastly, the desired atmospheric forcing field is cropped to the FVCOM grid using the latitude and longitude indices, and the interpolation coefficients and indices are used as follows to obtain the forcing field for FVCOM

(13) $$F_{\left(n,t\right)}=\sum_{j=1}^{4}\alpha_{\left(n,j\right)\times }X_{\left(n,j,t\right)},$$

where, for each time step ‘t’, F_(n,t)_ represents the forcing field at that time step for *n* = 1: number of nodes/cells, α_(n,j)_ denotes the nearest four [*j* = 1:4] coefficients surrounding the node/cell ‘n’ and X_(n,j,t)_ represents the atmospheric forcing field extracted at the nearest four [*j* = 1:4] indices surrounding the node/cell ‘n’ at time step ‘t’.

### Ice Nudge module

Sea ice freezing/melting is an important aspect of high latitude ocean circulation. During sea ice freezing/melting, there are momentum, heat, freshwater and salt exchanges across the sea ice-ocean interface. In order to resolve the ocean dynamics in the presence of sea ice, we have implemented a new module called ICENUDGE into FVCOM. Furthermore, this allows us to simulate the sensitivity of the PGIS melt to seasonally varying sea ice conditions [such as in [41]]. The module essentially uses the read-in sea ice concentration to determine where to modify the ocean surface fluxes. In the following sections, we present assumptions, equations and implementation of the module, and a description of the associated variables and parameters are provided in Tables 2 and 3.

#### Momentum flux

For momentum flux at the sea ice-ocean interface, we use a stress formulation that takes into account the velocity difference between the sea ice and the ocean. Following [16], the stress at the sea ice-ocean interface $\vec{\tau_{io}}$ is given by

(14) $$\vec{\tau_{io}}=c_{d}\rho_{w}\left|\vec{U_{i}}-\vec{U_{w}}\right|\left[\left(\vec{U_{i}}-\vec{U_{w}}\right)\text{cos}\theta +\hat{k}\times \left(\vec{U_{i}}-\vec{U_{w}}\right)\text{sin}\theta \right]\;.$$

Here, $\vec{U_{i}}$ is the sea ice velocity read from forcing, $\vec{U_{w}}$ is the ocean surface current velocity, $\rho_{w}$ is the density of seawater, $\hat{k}$ is the vertical unit vector, $c_{d}$ is the ocean drag coefficient and θ is the turning angle between geostrophic and surface currents. We combine the sea ice-ocean stress at the sea ice-covered part with the wind stress $\vec{\tau_{ao}}$ at the sea ice-free part as

(15) $$\vec{\tau_{s}}=\;A_{i}\;\vec{\tau_{io}}\;+\;\left(1\;-\;A_{i}\right)\vec{\tau_{ao}},$$

where, $\vec{\tau_{s}}$ is the surface stress used in FVCOM, and $0\;\leq \;A_{i}\;\leq \;1$ is the sea ice concentration read from forcing.

#### Thermodynamical flux to the ocean

*Assumptions*: Before presenting the detailed thermodynamics used in the module, we first state the important assumptions that we make:

• There is no snow.
• There is only basal melting and no lateral melting.
• The sea ice surface temperature is known and is set to the surface air temperature.
• The sea ice is fresh when calculating the conductive heat flux and freshwater flux.
• The mixed layer is the uppermost model layer.

*Fluxes due to basal melting*: Following [15], the heat flux at the sea ice-ocean interface can be estimated using a 2-equation approach as

(16) $$T_{f}=\;-\mu S_{w},$$

and,

(17) $$\rho_{i}m_{i}L_{i}=K_{0}\frac{T_{f}-\;T_{s}}{h_{i}}-\;\rho_{w}c_{w}c_{h}u^{*}\left(T_{f}-T_{w}\right)\;.$$

Here, $T_{f}\;$is the surface freezing temperature, μ is the ratio of the freezing point temperature to the salinity of the brine,$\;S_{w}$ is the salinity of the upper layer, $\rho_{i}\;$is the density of ice, $m_{i}\;$is the melting rate (positive for melting and negative for freezing), $L_{i}\;$is the latent heat of fusion of ice, $K_{0}$ is the thermal conductivity of fresh ice, $T_{s}\;$is the sea ice surface temperature, $h_{i}\;$is the sea ice thickness, $c_{w}\;$is the specific heat capacity of sea water, $c_{h}$ is the heat transfer coefficient, $u^{*}\;$is the friction velocity for ocean heat flux, and $T_{w}\;$is the water temperature of the upper layer.

Eq. (17) tells us that the basal melting or freezing (the first term) is determined by the sum of the conductive heat flux (the second term) and the turbulent heat flux from the ocean (the third term). In most cases, the conductive heat flux contributes to the basal freezing, and the turbulent heat flux from the ocean contributes to the basal melting. In the ICENUDGE module, we separate the melting and freezing processes when calculating the total heat flux entering (or leaving) the ocean surface as

(18) $$\rho_{i}m_{it}L_{i}=-\rho_{w}c_{w}c_{h}u^{*}\left(T_{f}-T_{w}\right),$$

and,

(19) $$\rho_{i}m_{ic}L_{i}=K_{0}\frac{T_{f}-\;T_{s}}{h_{i}}\;.$$

Here, $m_{it}$ is the basal melting rate due to the turbulent heat flux, $m_{ic}\;$is the basal freezing rate due to the conductive heat flux, and $m_{i}=\;m_{it}+\;m_{ic}$ . The net heat flux $FH_{\text{io}\;}$at the sea ice-ocean interface is given by

(20) $$FH_{\text{io}\;}=F_{\text{bot}\;}+F_{\text{hc}\;}+F_{con}+F_{\text{swthru}}\;.$$

Here, $F_{bot}=\rho_{w}c_{w}c_{h}u^{*}\left(T_{f}-T_{w}\right)$ is the latent heat flux due to basal melting, and it is negative when the ocean is losing heat while melting sea ice. $F_{hc}=c_{w}\rho_{i}m_{ic}(T_{f}-$ $T_{w}$) is the heat content of the melted water when using a 'virtual salt flux' approach [21]. $F_{con}=K_{0}\frac{T_{f}-T_{s}}{h_{i}}$ is the conductive heat flux. The last term in Eq. (20) is the shortwave radiation flux that passes through the sea ice [16] as

(21) $$F_{swthru}\;=I_{0}\exp\left(-\kappa_{i}h_{i}\right),$$

where,

(22) $$I_{0}=i_{0}\left(1-\;\alpha_{i}\right)F_{sw}\;.$$

Here, $I_{0}$ is the shortwave radiation flux penetrating into the sea ice, $i_{0}$ is a constant determining the fraction of penetrating shortwave radiation, $\alpha_{i}\;$is the albedo, $\kappa_{i}$ is the extinction coefficient, and $F_{sw}$ is the shortwave radiation incident at the surface.

We combine the net heat flux $F_{io}$ at the sea ice-covered part with the net heat flux from the atmosphere $FH_{ao}$ at the sea ice-free part as

(20) $$FH=A_{i}FH_{io}+\left(1-A_{i}\right)FH_{ao},$$

where, FH is the net surface heat flux used in FVCOM. In addition, the shortwave radiation flux FSW is also modified as

(21) $$FSW=A_{i}F_{swthru}+\left(1-A_{i}\right)F_{sw}\;.$$

We use the combined net surface heat flux and the combined shortwave radiation flux as boundary conditions to resolve the temperature equation and update the temperature in the model.

For the freshwater and salt fluxes, we use a 'virtual salt flux' approach [21] as

(22) $$F_{S}=-0.001\rho_{i}m_{it}\left(S_{w}-S_{i}\right),$$

where, $F_{S}$ is the virtual salt flux from basal melting, $S_{i}$ is the sea ice salinity read from forcing. We compute a transit salinity $S_{tmp}$ of the upper layer at the sea ice-covered portion with the virtual salt flux $F_{S}$ in a simplified way as

(23) $$S_{tmp}=S_{w}+\frac{F_{S}dt}{\rho_{w}D_{u}},$$

where, dt is the internal time step of integration, $D_{u}$ is the depth of the upper layer. Ideally, it is the mixed layer depth that should be used, however, here we currently simplify the mixed layer depth to the uppermost model layer depth.

*Fluxes due to sea ice freezing:* Unlike computing the melting-induced fluxes only at the sea ice covered part, we compute fluxes both due to basal freezing at the sea ice covered and frazil ice freezing at the sea ice free parts, based on the updated temperature and salinity of the upper layer. When sea ice is freezing, the heat loss of the upper layer cell is split into two parts: a sensible heat loss that cools the water to the surface freezing temperature, and a latent heat loss that buffers the ocean temperature to not go below the freezing temperature. Quantifying the amount of heat that is lost by this release of latent heat from freezing will tell us how much sea ice is being formed and hence how much brine is released into the water. For this purpose, we first compute the total amount of heat from the ocean that is available for sea ice freezing $F_{frzmlt}$ for both the sea ice free and sea ice covered ocean as

(27) $$F_{frzmlt}=\rho_{w}c_{w}\left(T_{w}-T_{f}\right)\;D_{u}/dt\;.$$

A negative $F_{frzmlt}$ indicates that there is more heat lost than the available sensible heat, and we use it to compute the amount of sea ice being formed via latent heat of fusion as

(28) $$\rho_{i}m_{it}L_{i}=\;F_{frzmlt}\;.$$

The corresponding virtual salt flux is given by Eq. (25) with $S_{i}=0.14\;S_{w}\;$being the salinity of the newly formed sea ice [38]. We sum the virtual salt fluxes from the basal melting and the sea ice freezing as a surface boundary condition to resolve the salinity equation and update the salinity field in FVCOM. The sensible heat loss of the surface cooling is treated in an implicit way by setting the temperature of the upper layer back to the freezing temperature where $F_{frzmlt}$ is negative.

Note that these fluxes updating the temperature and salinity fields are being computed at each model time step, and they should not be seen as two processes that happen after each other.

The sea ice concentration ($A_{i}$), sea ice thickness ($h_{i}$), bulk ice salinity ($S_{i}$) and sea ice velocity ($\vec{U_{i}}$) are prescribed at all nodes/cells at the desired forcing time step. Inside the Petermann Fjord, the sea ice-ocean stress, heat/salt flux due to freezing/melting of ice is set to zero for all internal nodes/cells beneath the PGIS. The required variables are derived from the coupled A4 ROMS-CICE run having 5 different ice categories, where A4 ROMS is coupled to the Los Alamos CICE (Community Ice CodE) sea ice model [16]. As the chosen model domain here is the Arctic-4 km (A4), the nesting interpolation method designed for A4 (described above) is adapted to extract the daily averaged ice nudge forcing variables at all internal and boundary nodes/cells for the entire duration of our simulation.

## Method validation

**Fig. 8:**
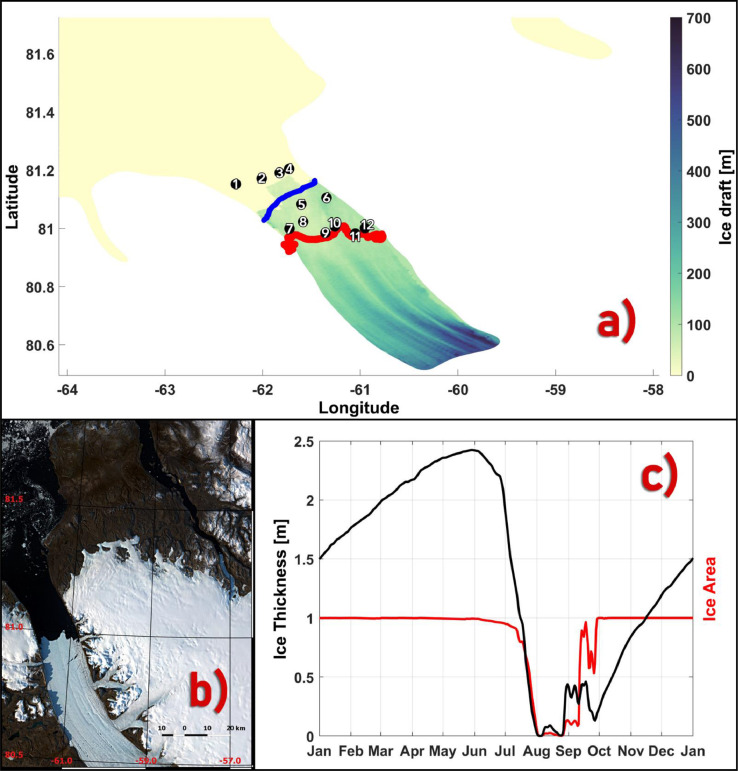
(a) The CTD station locations [numbered 1–12] sampled on September 04 during the 2019 Ryder expedition plotted over the model ice-draft (in meters). The calving front position (not corrected for coastline mismatch) from September 08, 2019 is overlaid in red. The overturning cross-section is highlighted in blue [see Section 6.2]. (b) 10 m Sentinel 2A imagery of the Petermann Glacier and fjord from September 08, 2019 showing a sea ice free fjord. (c) Mean (over stations 1–4) modelled sea ice area (red) and thickness (black) time series from Jan 01, 2016 –Jan 01, 2017. Longitude and latitude are expressed in °east and °north, respectively.

A realistic PGIS basal topography, a locally modified Petermann Fjord bathymetry which includes the inner sill ∼ 25 km from the GL, and a set of bias corrected far field ocean conditions with inter-annual variability driven by the tidal, sea ice and atmospheric conditions define the standard (reference) run of our setup. The ocean in the standard run is initialized from rest on July 01, 2014 00:00:00 UTC using the A4 temperature and salinity fields and run using time dependent forcings as described above. Output from the first 6 months of this simulation are discarded to allow sufficient time for the model to converge to a steady state solution. It is then restarted using the model solution on January 01, 2015 00:00:00 UTC, following which it is allowed to run until January 01, 2017 00:00:00 UTC using the time dependent forcings as described above. Additionally, another simulation, identical to the standard run but without the bias correction applied to the far field ocean conditions is run. The daily averaged modelled potential temperature (θ) and practical salinity (S) fields from these simulations from the Petermann Fjord on September 04, 2016 are compared against CTD measurements taken on September 04, 2019 during the Ryder 2019 expedition [[19], Fig. 8(a)]. Aside from a difference in timestamp, it is also important to note that stations 1 –4 were sampled near the fjord entrance which is ∼26 km from the September 08, 2019 mean calving front position [Fig. 8(a)] and ∼7 km from the BedMachine v3 derived mean model calving front position [Fig. 8(a)] which represents a period prior to the 2012 and 2010 calving events [8,31]. Moving landward, stations 5 –12 were sampled closer to the calving front position as of September 08, 2019 [Fig. 8(a)], however, in our model, these correspond to sub ice shelf temperature and salinity profiles, and therefore, they are only used to compare the modelled θ-S signature of the deeper AW in conjunction with previously reported values of the same from within the fjord and ice shelf cavity (see below). Furthermore, inferring from the observed θ-S profiles from the Lincoln sea area [30,46], the A4 northern boundary water column remains relatively colder and fresher even after the applied bias correction (for reasons that we will explore below).

### Modelled summer θ-S profile evaluation from the Petermann Fjord

**Fig. 9:**
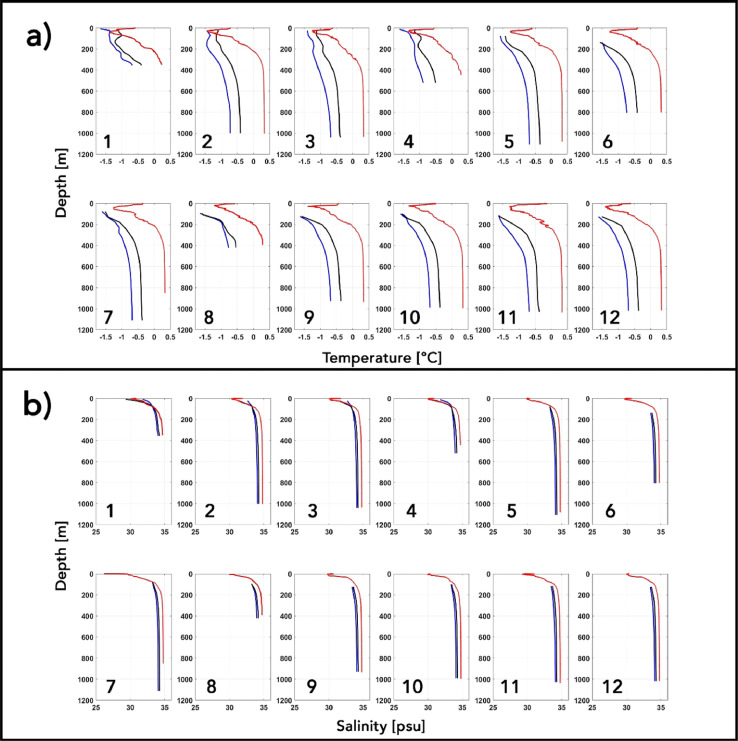
Observed (red), biased (blue) and bias corrected (black) modelled profiles of (a) temperature and (b) salinity at the sampled (1–12) CTD locations.

A cold (−1.5 °C < θ < −0.5 °C) and fresh (30 psu < *S* < 32 psu) layer of Polar Water (PW) of Arctic origin is observed [Fig. 9(a) and (b)] occupying the upper ∼45–50 m portion of the water column, where near surface shortwave warming in the absence of sea ice cover in the fjord [Fig. 8(b)] likely raises the temperature to ∼ −0.5 °C and freshening likely driven by glacial and terrestrial surface runoff lowers the salinity to ∼30 psu. While these samples correspond to a snapshot of the state of the fjord, the observed θ-S ranges for the different water masses are in good agreement with previously reported values [[13], [22], [46]]. A range of differing factors need to be considered when comparing the above signature with that of the model. In addition to the ones suggested above, these include, but are not limited to, the prevailing sea ice conditions in the fjord, local atmosphere and surface freshwater runoff. The model is run without any river runoff sources and while differences in local atmospheric state are relevant, its investigation in this context falls beyond the scope of our work. For stations 1 –4, we noticed that both the biased and bias corrected modelled near surface waters do not warm as much as seen in the observation which could be attributed to the presence of a shallow and thin (aice = 0.25 and hice = 0.5 m) modelled sea ice cover over the stations [Fig. 8(c)]. This thin local summer sea ice cover is also likely to undergo enhanced melting in the presence of a bias corrected warmer water column [Fig. 7(b), Fig. 9(a)] resulting in an upper water column that is fresher and more in agreement with the observed profiles than the biased run at similar depths [Fig. 9(b)]. Up to depths of ∼80 m, unlike the biased θ-S profiles, the bias corrected temperature profiles from the model are seen to intersect with the observed temperatures [Fig. 9(a)] and a good agreement is seen for the salinity profiles as well which overlap the observed profile [Fig. 9(b)]. Below this depth, the observed θ-S profiles are seen to depart towards a warmer and saltier AW when compared to the modelled profiles [Fig. 9(a) and (b)], which stems from the upstream A4 AW boundary conditions.

The sub ice shelf modelled θ-S profiles from stations 5 –12 are not influenced by surface processes [Fig. 9(a) and (b)] and a similar discrepancy between the observed and modelled AWs at depth (as described above for stations 1 –4) is seen underneath the ice shelf cavity [Fig. 9(a) and (b)]. As opposed to a cold bias that is seen in the A4 AW at the northern boundary, a strong subsurface ocean warming trend is reported in the Nares Strait for the period between 2003 and 2015 and therefore, the AW that spill over the outer sill into the fjord and underneath the ice shelf cavity have also become warmer [30,46]. While the bias correction method adopted here helps to bridge the gap [Fig. 9(a) and (b)], these AW at the northern boundary still remain relatively colder and fresher when compared to observed signature, resulting in an underestimation of the θ-S signature in the Nares Strait and Petermann Fjord and ultimately the heat transport to the PGIS and the basal melt rates.

### Turbulent heat transfer coefficient (Γ_T_) sensitivity experiments

**Fig. 10:**
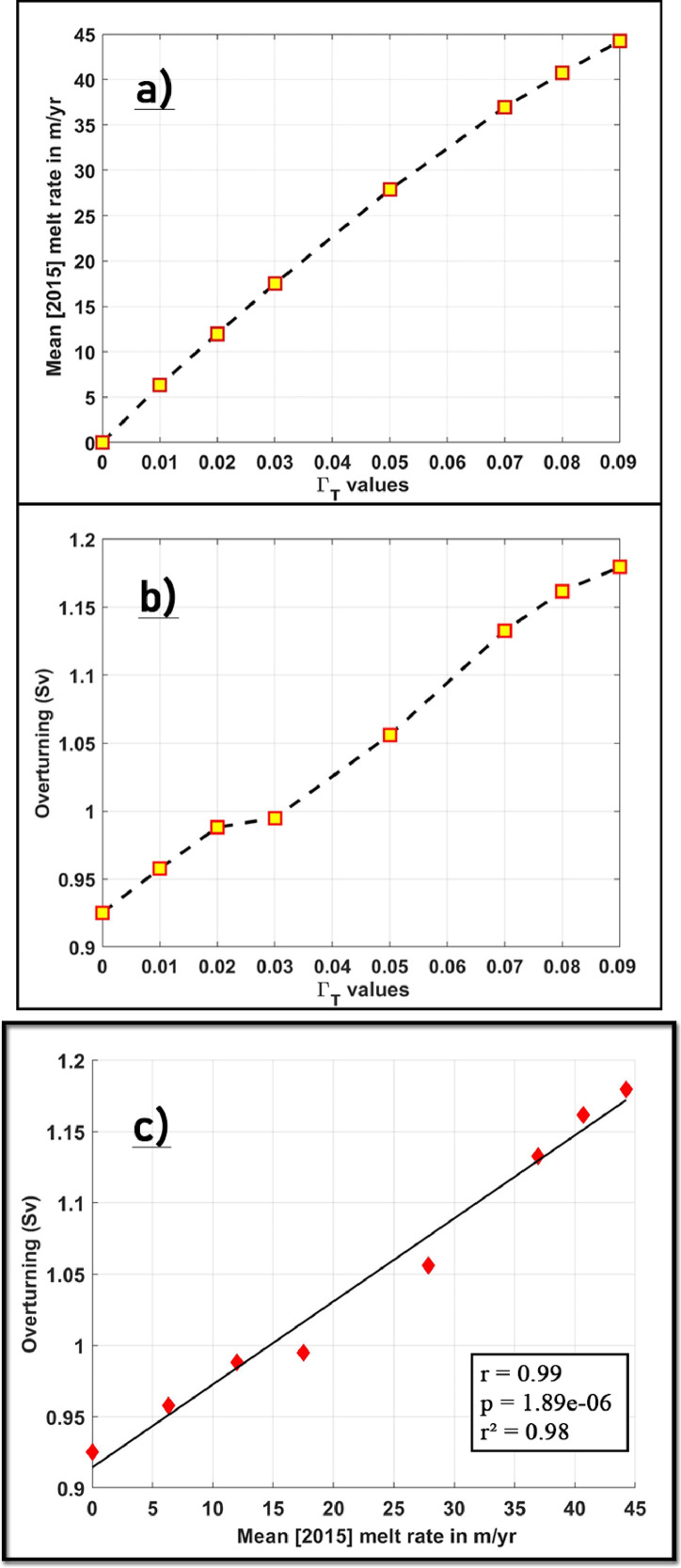
Modelled mean (2015) (a) basal melt rates in m/yr averaged over the entire PGIS base and (b) amplitude of the overturning circulation in Sverdrup (Sv), expressed as a function of Γ_T_ values. (c) The annual mean overturning amplitude in Sv [from (b)] versus the annual mean area averaged melt rates [from (a)]. The overturning circulation is calculated through a cross-section spanning the entire width of the fjord and located near the calving front [highlighted in blue in Fig. 8(a)]. The least-mean-squares regression line is indicated in black, and the corresponding correlation (r), p-value (p) and coefficient of determination (r^2^) are indicated in the lower right corner.

Following the six-month spin-up period of our standard run, the heat flux through the ice shelf-ocean boundary layer is scaled by varying the turbulent heat transfer coefficient (Γ_T_) in a series of 8 × 1-year long (January 01, 2015 00:00:00 UTC – January 01, 2016 00:00:00 UTC) sensitivity experiments. Results from the experiments are shown in Fig. 10(a), where modelled basal melt rates are seen to increase with an increasing Γ_T_, generating annual mean basal melt rates averaged over the entire PGIS base that range from 0 –45 m/yr for 0 ≤ Γ_T_ ≤ 0.09 [Fig. 10(a)]. However, we notice that the slope becomes weaker as Γ_T_ increases, and therefore, the melt rate does not increase linearly with Γ_T_, indicating the likelihood of a feedback mechanism for the PGIS and Fjord system. We argue that the non-linear relationship seen here results from a positive feedback, where stronger melting due to an increase in Γ_T_ strengthens the overturning circulation inside the fjord [Fig. 8(a), Fig. 10(b)] and likely increases the heat transport toward the ice shelf (not shown here). For PGIS, under the configuration described here, we see considerable overturning without any melt (Γ_T_ = 0) and an increase of it as melting increases (Γ_T_  > 0). The overturning intensifies in the mid-value Γ_T_ range [0.03 < Γ_T_ ≤ 0.07], while the curve saturates with weaker slopes for the high and low Γ_T_ values [Fig. 10(b)]. Furthermore, the annual mean overturning amplitude and the annual mean area averaged melt rates are seen to closely follow each other [Fig. 10(c)], where the overturning circulation within the PGIS cavity increases linearly with the area averaged PGIS melt rate. Such a melt –cavity circulation feedback has also been reported for ice shelves in the Amundsen Sea [24]. However, a deeper investigation of these mechanisms falls beyond the scope of this article.

Wilson et al. [48] estimate PGIS melt rates that range from ∼ 50 m/yr near the GL decreasing to ∼ 10 m/yr seaward of it. Therefore, a Γ_T_ = 0.05 is chosen for our standard run which generates a mean melt rate of ∼ 28 m/yr [Fig. 10], broadly consistent with the reported melt rate estimates described above. However, as the A4 ocean boundary conditions remain unchanged, the AW entering the fjord are still colder and fresher when compared to the observed values and the θ-S signature of the resulting AW-PGIS meltwater mixture that gets formed is likely to retain this characteristic as well. Furthermore, by varying Γ_T_ to scale the heat flux, we demonstrate a range of melt rates that can be simulated by the model for a given set of far-field boundary conditions, and adopt a value of Γ_T_ specific to the A4 ocean boundary conditions that are currently in place. Therefore, for studies focusing on investigating the sensitivity of PGIS to future warming of AW using the model configuration described here, it is important to note that the higher value of Γ_T_ used here relates to the relatively colder and fresher upstream A4 ocean boundary conditions, and would likely lead to a higher sensitivity of the model to future warming scenarios. In these studies, a depth dependent bias correction can first be applied (such as in [40]) to the upstream A4 θ-S fields to generate boundary conditions that fit the observed profiles. The chosen Γ_T_ value would then be consistent with the present conditions and additional sensitivity experiments that differ in the applied ocean forcing can be systematically performed.

### Modelled AW-PGIS meltwater mixture characteristics

**Fig. 11:**
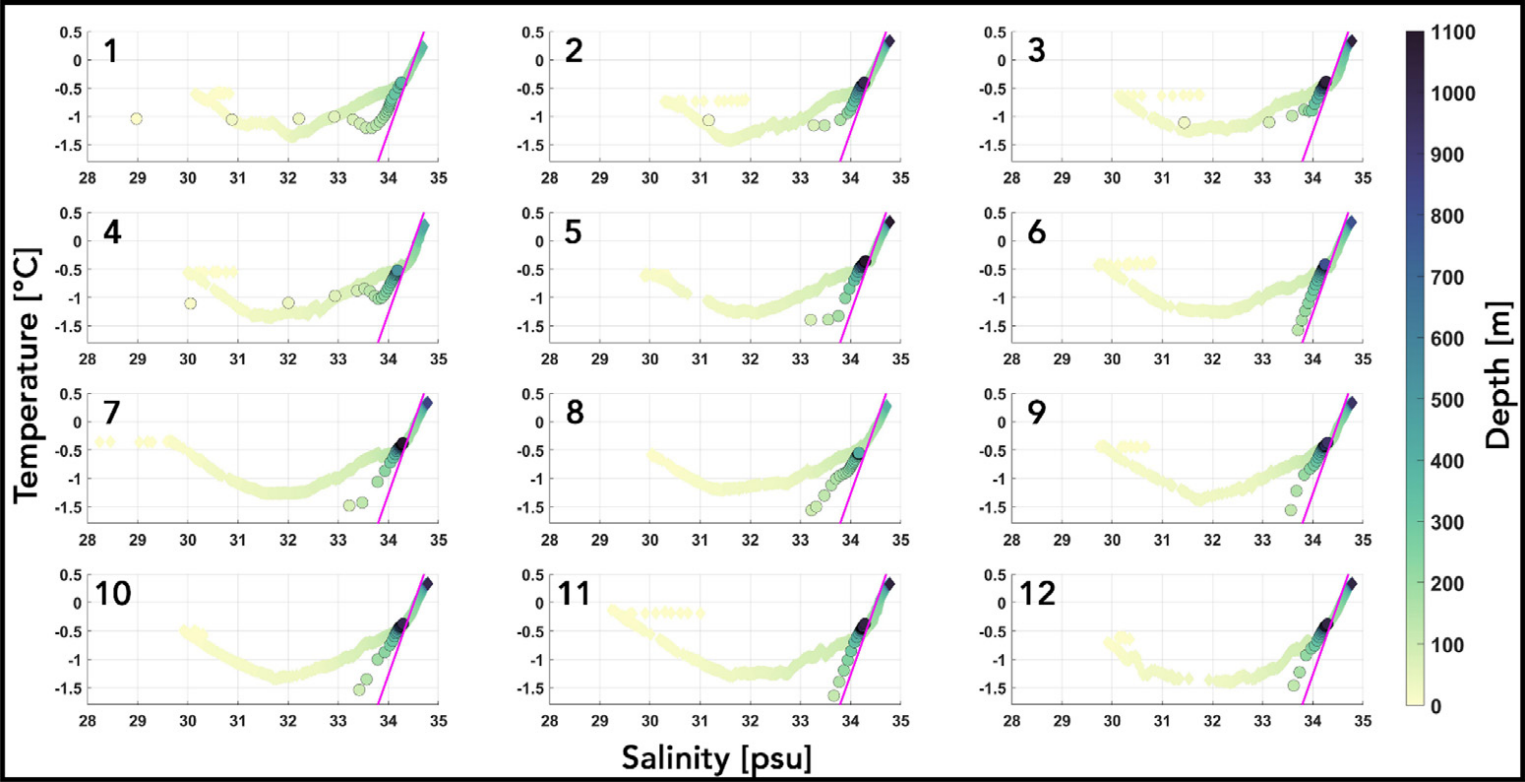
Observed (diamond) and bias corrected modelled (circles) temperature vs salinity profiles at the sampled (1–12) CTD locations color coded with depth (in meters). The overlaid magenta line indicates the AW-PGIS meltwater mixing (Gade) line.

Both the observed and bias corrected profiles from the model for all the stations are represented in a θ-S space together with a straight line that has a slope of 2.5 °C/psu [Fig. 11]. This line is referred to as the Gade line [10] and depicts a θ-S relation that results from glacial ice being melted by ocean water. Here, it can be seen as the AW –PGIS meltwater mixing line where its slope represents the mixing of these two end member water masses. The AW is characterized as the bias corrected θ-S signature from the model [Fig. 9 (a) and (b)] at the model mean GL depth of 600 m. Here, these correspond to AW having θ = θ_AW_ and *S* = S_AW_ [see Table 4]. The basal melting of glacial ice along the PGIS base from this warm and saline ambient AW will draw energy in the form of latent heat transfer from the AW to melt the ice. This fresh PGIS meltwater being released will blend with the ambient AW. The path in θ-S space resulting from latent heat loss and freshening and the mixing of AW with PGIS meltwater is defined by temperature (θ_g_) and salinity (S_g_) constrained by the Gade line [10,20], such that

(29) $$\theta_{g}\left(S_{g}\right)=\theta_{0}+\frac{L}{C_{o}}\left(1-\frac{S_{0}}{S_{g}}\right),$$

where, θ_o_ = θ_AW_ and S_o_ = S_AW_ [see Table 4], respectively, are the temperature and salinity of the warm and saline ambient AW that melts the PGIS from below. L denotes the latent heat of fusion of ice and C_o_ is the specific heat capacity of seawater having salinity = S_AW_, temperature = θ_AW_ and pressure = 588 dbar [see Table 4]. First, we noticed that all observed profiles [Fig. 11] below a depth of 130 m fit the Gade line indicating that these are formed as a result of a clear mix between the two end member water masses, namely the AW and PGIS meltwater. They are then seen to depart from this line higher up in the water column [Fig. 11] towards a shallower slope where freshening strengthens and the latent heat loss weakens. This suggests that the ambient AW is entraining one or more fresher water mass without spending the energy required for melting the PGIS. Terrestrial and/or glacial runoff [43] entering either as subglacial discharge flux across the GL or as surface glacial meltwater transported to the ocean across the terminus via rivers along with sea ice melt are the likely freshwater sources. The θ-S profile of the upper water column from the fjord resembles the Hall Basin [19] with past hydrographic campaigns [[13], [22]] reporting similar results. Such an AW-freshwater mixing in the upper water column, therefore, is not limited to the fjord, but can also occur in the greater Hall Basin-Nares Strait-Arctic Ocean region. A clear distinction is seen between the modelled θ-S profiles taken from slightly north of the model calving front position (stations 1 – 4) and those that correspond to sub ice shelf profiles (stations 5 – 12). For stations 1 – 4, deep water masses (below 170 m) are seen to fall on the Gade line with shallower waters showing a similar departure of the profile away from the Gade line as seen in the observations [Fig. 11]. θ-S profiles from stations sampled close to the northeastern fjord wall (around station 4) and slightly north of the calving front position in 2009 [22] exhibited a similar runoff-point (see [43] for definition) depth of 185 m. Similar diagnostics from the fjord report this depth to range between 135–230 m [22,46] which agrees well with our modelled value. In the absence of terrestrial and glacial runoff sources in our standard run, such departures from the Gade line could be driven by sea ice melt. It could also likely be representative of the mixing of AW or the AW-PGIS meltwater mixture with the cold (near freezing) and fresh winter surface waters that are abundant in the fjord and strait. In the ice shelf cavity (stations 5 – 12), without runoff, the modelled θ-S profile does not exhibit such strong departures; nearly fitting the Gade line up to the corresponding ice shelf-ocean interface depth [Fig. 11]. Through-ice CTD data gathered from the central PGIS channel 3 km from the GL [46] showed a similar distribution. We see a better fit for the modelled sub ice shelf stations along the right flank, which could likely be due to the presence of a strong outflow of meltwater that exits the fjord keeping the eastern Greenland coast to its right (not shown here), however, further discussions would require a thorough investigation of the fjord circulation which falls beyond the scope of this article.

**Table 2:** Description of the variables used in the Ice Nudge module.

Variable (Unit)	Description	Type
$\vec{\tau_{io}}\;\left(Nm^{-2}\right)$	Stress at the sea ice-ocean interface	Prognostic
$\vec{\tau_{ao}}\;\left(Nm^{-2}\right)$	Stress at the atmosphere-ocean interface	Read from forcing
$\vec{\tau_{s}}\;\left(Nm^{-2}\right)$	Stress at the ocean surface	Prognostic
$\vec{U_{i}}\;\left(ms^{-1}\right)$	Sea ice velocity	Read from forcing
$\vec{U_{w}}\;\left(ms^{-1}\right)$	Ocean surface velocity	Prognostic
$A_{i}$	Sea ice concentration	Read from forcing
$S_{i}\;\left(\text{PSU}\right)$	Sea ice salinity	Read from forcing when ice is melting. Prognostic when ice is freezing
$S_{w}\;\left(\text{PSU}\right)$	Salinity of the uppermost layer	Prognostic
$h_{i}\;\left(m\right)$	Sea ice thickness	Read from forcing
$T_{f}\;\left({\;}^{\circ }C\right)$	Surface freezing temperature	Prognostic
$T_{s}\;\left({\;}^{\circ }C\right)$	Sea ice surface (air) temperature	Read from forcing
$T_{w}\;\left({\;}^{\circ }C\right)$	Temperature of the uppermost layer	Prognostic
$m_{i}\;\left(ms^{-1}\right)$	Melting rate	Prognostic
$FH_{io}\;\left(Wm^{-2}\right)$	Net heat flux at the sea ice-ocean interface	Prognostic
$F_{bot}\;\left(Wm^{-2}\right)$	Latent heat flux due to basal melting	Prognostic
$F_{hc}\;\left(Wm^{-2}\right)$	Heat content of the melted water	Prognostic
$F_{con}\;\left(Wm^{-2}\right)$	Conductive heat flux at the sea ice-ocean interface	Prognostic
$F_{swthru}\;\left(Wm^{-2}\right)$	Shortwave radiation flux penetrating the sea ice	Prognostic
$F_{sw}\;\left(Wm^{-2}\right)$	Incident shortwave radiation flux	Read from forcing
$FH\;(Wm^{-2})$	Net heat flux at the ocean surface	Prognostic
$FSW\;(Wm^{-2})$	Shortwave radiation flux at the ocean surface	Prognostic
$F_{S}\;\left(\text{kg}m^{-2}s^{-1}\right)$	Virtual salt flux	Prognostic
$S_{tmp}\;\left(\text{PSU}\right)$	Transit salinity of the uppermost layer	Prognostic
$D_{u}\;\left(m\right)$	Depth of the uppermost layer	Prognostic
$d_{t}\;\left(s\right)$	Time step of integration	Preset
$F_{frzmlt}\;\left(Wm^{-2}\right)$	Available heat flux for sea ice freezing	Prognostic

**Table 3:** Description of the parameters used in the Ice Nudge module.

Parameter	Value	Description
$c_{d}$	0.0055	Ocean drag coefficient
θ	0	Turning angle between geostrophic and surface current
$\rho_{w}$	$1026\;\text{kg}m^{-3}$	Density of sea water
$\rho_{i}$	$917\;\text{kg}m^{-3}$	Density of ice
μ	$0.054^{\circ }\text{CPS}U^{-1}$	Ratio of the freezing point temperature to the salinity of the brine
$K_{O}$	$2.03\;W{m^{-1}}^{\circ }C^{-1}$	Thermal conductivity of fresh ice
$L_{i}$	$3.34\;\times 10^{5}\;\text{Jk}g^{-1}$	Latent heat of fusion of ice
$c_{w}$	$4218\;J^{\circ }C^{-1}kg^{-1}$	Specific heat capacity of sea water
$c_{h}$	0.006	Heat transfer coefficient
$u^{*}$	$\max\left(\sqrt{\frac{\left(\vec{\tau_{io}}\right)}{\rho_{w}}},\;0.005\right)ms^{-1}$	Friction velocity
$k_{i}$	$1.4\;m^{-1}$	Extinction coefficient
$i_{0}$	0.7	Fraction of penetrating shortwave radiation
$\alpha_{i}$	0.7	Ice albedo

**Table 4:** Description of the parameters used in the method validation section.

Parameter	Value	Description
$\Gamma_{T}$	0−0.09	Non-dimensional heat-transfer coefficient
$\theta_{AW}$	$-0.5^{\circ }C$	Bias corrected AW potential temperature at the model mean GL depth of 600 m
$S_{AW}$	34.3PSU	Bias corrected AW salinity at the model mean GL depth of 600 m
L	334kJ/kg	Latent heat of fusion of ice
$C_{o}$	3.97kJ/kg/C	Specific heat capacity of seawater

## Summary

Here, we present a nested, high-resolution unstructured grid, 3-D ocean-sea ice-ice shelf setup based on the Finite Volume Community Ocean Model (FVCOM) which includes a realistic ice shelf basal topography [4,^5^,51], to study the Petermann Glacier Ice Shelf (PGIS) and fjord system. Lacking bathymetric measurements under the PGIS, we systematically adjusted and smoothed the topographic variables (namely h, zisf and WCT) in this region. We implemented further changes to incorporate bathymetric features that were revealed by Operation Ice Bridge aerogravity [45] including a dynamically important inner sill ∼ 25 km from the GL. These modifications resulted in a physically plausible and numerically robust representation of the topography underneath the PGIS. We defined a standard (reference) run which utilizes the aforementioned topography together with bias-corrected far-field ocean conditions derived from a nested 4-km pan-Arctic (A4) ROMS model [12] and which allows all inter-annual variability to be attributed to the tidal or local sea ice or atmospheric conditions that are derived from a suite of high-resolution regional models.

Bias correction that we applied to the northern A4 boundary conditions resulted in noticeable improvements. However, the AW θ-S signature remains relatively colder and fresher when compared to observations from the fjord. To compensate for the underestimation of basal melt rates, we ran a series of sensitivity experiments to derive a value for the turbulent heat transfer coefficient (Γ_T_) which is appropriate for the given upstream A4 boundary conditions, following which, a Γ_T_ of 0.05 is chosen which generated a simulated mean melt rate that largely agrees with the estimated figures reported by Wilson et al. [48]. With these experiments, we demonstrated a broad range of basal melt rates that can be generated by the model for a given set of boundary conditions. Furthermore, we showed that the non-linear response of the PGIS basal melt to an increasing Γ_T_ is due to a self-amplifying feedback mechanism, wherein, melt induced overturning strengthens as Γ_T_ increases, likely increasing the transport of warm and saline AW in the PGIS cavity which further enhances the basal melting [as shown by Jourdain et al. [24] for ice shelves in Antarctica]. Modelled profiles of temperature and salinity from the fjord depicted in a θ-S space show good agreement with reported ranges of runoff-point depths indicating that the model is able to accurately depict the relevant ice-ocean interaction mechanisms under the existing conditions. The most noticeable difference is driven by the relatively cold and fresh upstream A4 boundary conditions, however, their contributions with regard to the ice shelf thermodynamics and the PGIS basal melt rates are largely compensated for by the higher Γ_T_ value derived from the sensitivity experiments. Here, it is important to note that this value is defined for the A4 boundary conditions that are described above. The θ-S fields still retain the bias, and therefore, we suggest applying a depth-dependent bias correction to the upstream A4 θ-S fields [such as in [40]] to match the present-day observational values, and calibrating Γ_T_ against a series of bias corrected control experiments, before using the model setup to perform sensitivity studies that depart from the ocean conditions in the control run. Presence of runoff sources, although not included in the definition of the standard run, can be added as an additional component as part of a wide range of transient experiments that can be created using our model by branching off from the standard run.

The intention is that following the methods and guidelines described here, the user should be able to create a similar setup, or modify it to fit other applications of ice shelf-ocean interactions. Furthermore, the smoothed bathymetry dataset along with the water column thickness and ice-draft datasets can be used in other σ-coordinate numerical ocean/ice shelf-ocean modeling application that aims to investigate the Petermann Fjord region. Lastly, by making use of all the input, nesting and forcing files, it should be possible to replicate the simulation results that are presented here.

## Declaration of Competing Interest

The authors declare that they have no known competing financial interests or personal relationships that could have appeared to influence the work reported in this paper.
